# Beyond Genetics: The Role of Metabolism in Photoreceptor Survival, Development and Repair

**DOI:** 10.3389/fcell.2022.887764

**Published:** 2022-05-18

**Authors:** Joseph Hanna, Luke Ajay David, Yacine Touahri, Taylor Fleming, Robert A. Screaton, Carol Schuurmans

**Affiliations:** ^1^ Sunnybrook Research Institute, Biological Sciences, Toronto, ON, Canada; ^2^ Department of Laboratory Medicine and Pathobiology, University of Toronto, Toronto, ON, Canada; ^3^ Department of Ophthalmology and Vision Sciences, University of Toronto, Toronto, ON, Canada; ^4^ Department of Biochemistry, University of Toronto, Toronto, ON, Canada

**Keywords:** photoreceptor development, retinal degeneration, Müller glia regeneration, glycolysis, mitochondria, oxidative phosphorylation, metabolic reprogramming

## Abstract

Vision commences in the retina with rod and cone photoreceptors that detect and convert light to electrical signals. The irreversible loss of photoreceptors due to neurodegenerative disease leads to visual impairment and blindness. Interventions now in development include transplanting photoreceptors, committed photoreceptor precursors, or retinal pigment epithelial (RPE) cells, with the latter protecting photoreceptors from dying. However, introducing exogenous human cells in a clinical setting faces both regulatory and supply chain hurdles. Recent work has shown that abnormalities in central cell metabolism pathways are an underlying feature of most neurodegenerative disorders, including those in the retina. Reversal of key metabolic alterations to drive retinal repair thus represents a novel strategy to treat vision loss based on cell regeneration. Here, we review the connection between photoreceptor degeneration and alterations in cell metabolism, along with new insights into how metabolic reprogramming drives both retinal development and repair following damage. The potential impact of metabolic reprogramming on retinal regeneration is also discussed, specifically in the context of how metabolic switches drive both retinal development and the activation of retinal glial cells known as Müller glia. Müller glia display latent regenerative properties in teleost fish, however, their capacity to regenerate new photoreceptors has been lost in mammals. Thus, re-activating the regenerative properties of Müller glia in mammals represents an exciting new area that integrates research into developmental cues, central metabolism, disease mechanisms, and glial cell biology. In addition, we discuss this work in relation to the latest insights gleaned from other tissues (brain, muscle) and regenerative species (zebrafish).

## Introduction

Visual impairment has reached crisis levels, afflicting 2.2 billion people globally (World Health Organization—2019). While one billion of these individuals have lost their sight due to correctable or treatable problems (e.g., cataracts, lack of corrective lenses), the remaining half require interventions that either do not yet exist or have limited efficacy. Age-related macular degeneration (AMD) is a leading cause of vision loss and ultimately of legal blindness (i.e., less than 20/200 vision), affecting ∼1/30 individuals as they age, or 196 million people worldwide (Wong et al., 2014). In AMD, cone photoreceptors that are concentrated in the central macula degenerate first, leading to a loss of central, high acuity vision. Of the two types of AMD, wet or exudative AMD is diagnosed in ∼10% of patients and is associated with neovascularization that can be treated with anti-VEGF therapies. Conversely, neovascularization does not occur in dry, atrophic AMD, and thus currently has no treatment options. Hereditary retinal dystrophies also lead to photoreceptor cell death and vision loss, and while they are comparatively rare (∼1/4,000), these disorders are devastating as they often manifest at a younger age. For instance, Retinitis Pigmentosa (RP) begins with a loss of peripheral and night vision due to rod photoreceptor death in young adulthood, and evolves to include loss of cone photoreceptors and legal blindness (Sahel, 2014).

One strategy to restore vision in these patients is to replace lost photoreceptors. Currently, several approaches are being developed, such as transplanting photoreceptors directly, or grafting retinal pigment epithelial (RPE) cells, which prevent photoreceptor death. However, there are growing concerns with these approaches that include the risk of tumor formation, the need for immunosuppression, and ethical and supply concerns regarding cellular source. In addition, the design of gene therapy approaches to halt the progression of vision loss is challenging because for some diseases, including RP, causative mutations occur in hundreds of genes, making it difficult to design an effective single gene therapy. Indeed, the single Food and Drug Administration (FDA)-approved adenovirus gene therapy to treat RP targets a single mutation in RPE65, and thus is not applicable to the majority of RP patients (Russell et al., 2017). An alternative approach would be an exogenous or endogenous repair strategy to replace degenerating photoreceptors, which could work regardless of the underlying genetic drivers or cause. Even though the mammalian retina is devoid of active stem cells to replace photoreceptors that have died (Wilken and Reh, 2016), Müller glia have regenerative capacity in certain cold-blooded vertebrates, such as teleost fish, prompting investigators to search for ways to activate their latent stem cell potential in the mammalian retina.

An emerging theme is the important role that metabolism plays in sustaining retinal health at the cellular and tissue level, and the contributions of metabolic disorders to neurodegenerative disease. Metabolic syndrome is the deleterious systemic changes that develop as a consequence of high fat/sugar/salt diets, including insulin resistance and the build-up of “bad” low-density lipoprotein (LDL) cholesterol. Importantly, metabolic syndrome is also associated with accelerated aging and neurodegenerative disease, including in the retina (Roddy, 2021). However, metabolism can be reprogrammed, and several studies have reported that healthy caloric restriction can slow down cellular aging and increase lifespan (Finkel, 2015). These studies reflect the intrinsic ability of cells to alter their bioenergetics to sustain tissue homeostasis. Notably, metabolic reprogramming also occurs during neural cell differentiation (Iwata and Vanderhaeghen, 2021), and in the glial response to neurodegeneration (Afridi et al., 2020). Here, we will discuss current knowledge of the role that cellular metabolism plays in sustaining photoreceptor survival, in guiding photoreceptor cell fate decisions during retinal development, and as a factor to be considered for the design of regenerative strategies, especially Müller glial mobilization.

## An Introduction to Cellular Metabolism

### Central Bioenergetic Pathways

**FIGURE 1:**
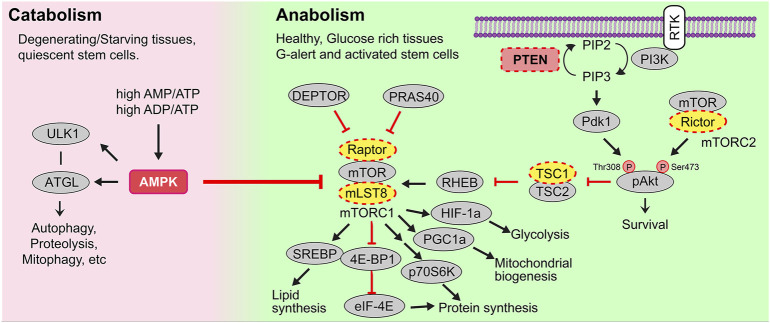
Catabolic and anabolic pathways. Catabolic or energy consuming processes (e.g., autophagy, proteolysis, mitophagy, etc) are triggered by AMPK. AMPK is activated in conditions of starvation when there are high AMP/ATP and ADP/ATP ratios, and in degenerating tissues or quiescent stem cells. Anabolic or energy building processes (e.g., glycolysis, mitochondrial biogenesis, mitochondrial fission, lipid synthesis) are controlled by growth factor signals, including those that bind receptor tyrosine kinases (RTK). Multiple downstream signals are activated by ligand-bound RTKs including the phosphoinositide-3-kinase (PI3K). PI3K activates the secondary messenger PIP3, a step that PTEN reverses. PIP3 then activates Akt and mTORC1, the latter a metabolic regulator that induces lipid and protein synthesis to drive cellular growth.

Metabolism refers to the balance of catabolic pathways that convert nutrients or macromolecules to energy (i.e., ATP) and anabolic pathways that synthesize macromolecules by consuming energy and nutrients (Figure 1). While catabolism is generally associated with starvation and the breakdown of muscle and fat to generate ATP, catabolic processes also predominate in degenerating tissues and in quiescent stem cells (Purohit and Dhawan, 2019). Conversely, anabolism occurs in healthy, metabolically active cells to support the biosynthesis of proteins, nucleic acids and lipids that are needed for cell growth and division**.** There are two main bioenergetic pathways–glycolysis, which occurs in the cytosol, and oxidative phosphorylation (OXPHOS), which occurs in the mitochondria.

### Glycolysis

**FIGURE 2:**
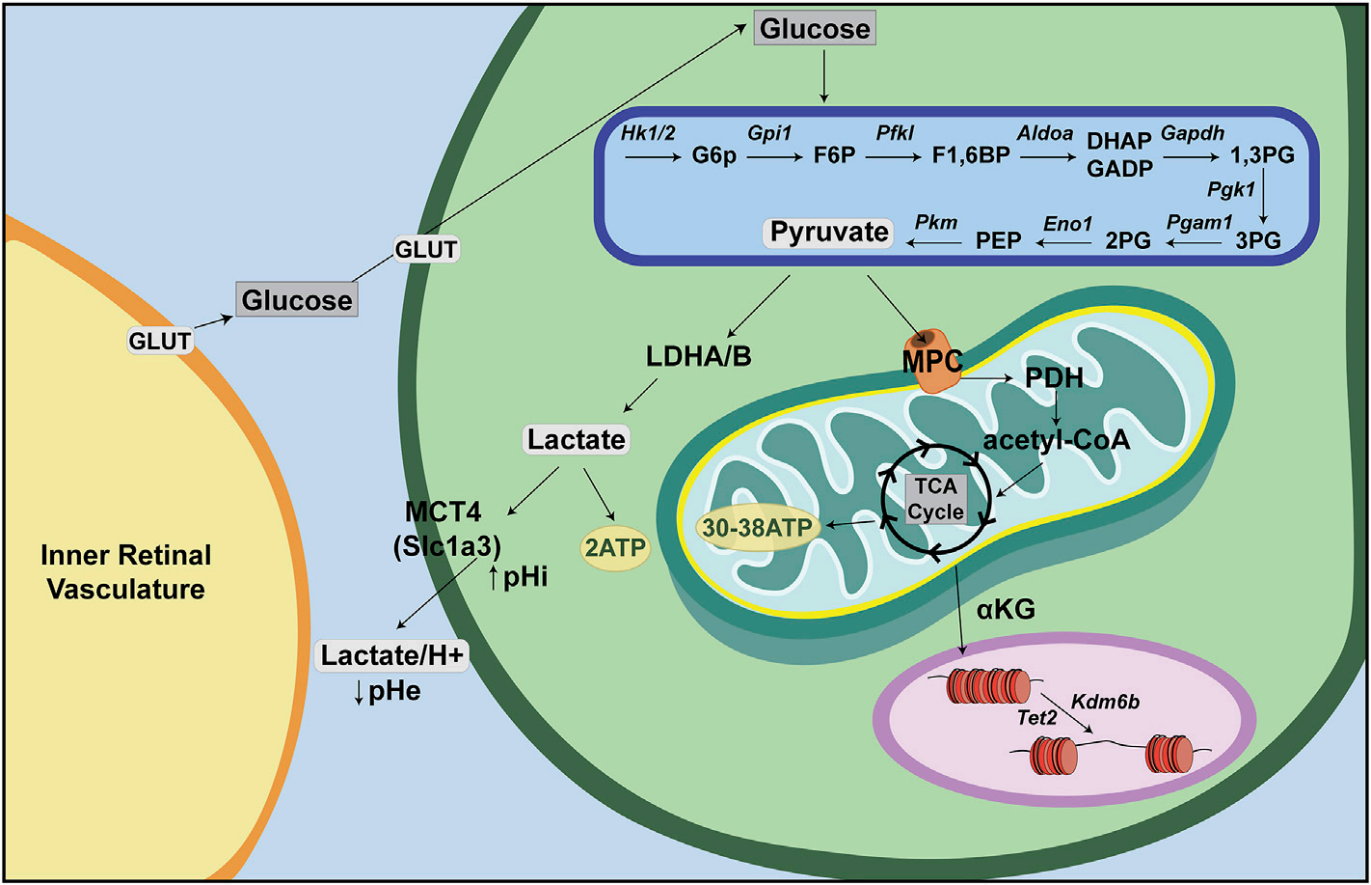
Energy metabolism—a retina-centric view. Glucose is supplied to inner retinal cells through the inner retinal vasculature and to photoreceptors through the choroidal vasculature. Glucose then enters retinal cells through GLUT transporters and is metabolized in anaerobic conditions through a multistep process called glycolysis. Glycolysis produces pyruvate, which can be converted to lactate and transported out of the cell *via* lactate/H^+^ symporters (Slc16a). In aerobic conditions, pyruvate is instead shuttled into the mitochondria where it is converted to acetyl-CoA to feed the tricarboxylic acid (TCA) cycle (or Krebs cycle). Acetyl-CoA is oxidized in a series of steps to make ∼30–38 ATP *via* oxidative phosphorylation (OXPHOS).

Glucose is the major nutrient and cellular fuel for ATP production. Glucose enters the cellular cytoplasm *via* one of 14 glucose transporters (GLUT1-14), now called solute carrier family 2a (SLC2A) transporters (Figure 2). Once in the cytoplasm, glucose is broken down to pyruvate *via* glycolysis, a multi-enzymatic cascade that does not require O_2_ and which generates two ATP per molecule of glucose. In anaerobic conditions, pyruvate is then converted to lactate and exported out of the cell along with a H^+^ ion by lactate/H^+^ symporters [SLC16A1, previously called monocarboxylate transporter 1 (MCT1), or SLC16A, previously MCT4], with a resultant decrease in extracellular pH (pHe) and increase in intracellular pH (pHi) (Oginuma et al., 2020). Notably, aerobic glycolysis, or Warburg metabolism, refers to glycolysis that occurs even in the presence of O_2_, which is a phenotype often associated with tumor cells, but also seen in non-cancerous cells, including some retinal cells, as discussed further below.

### Mitochondrial OXPHOS

**FIGURE 3:**
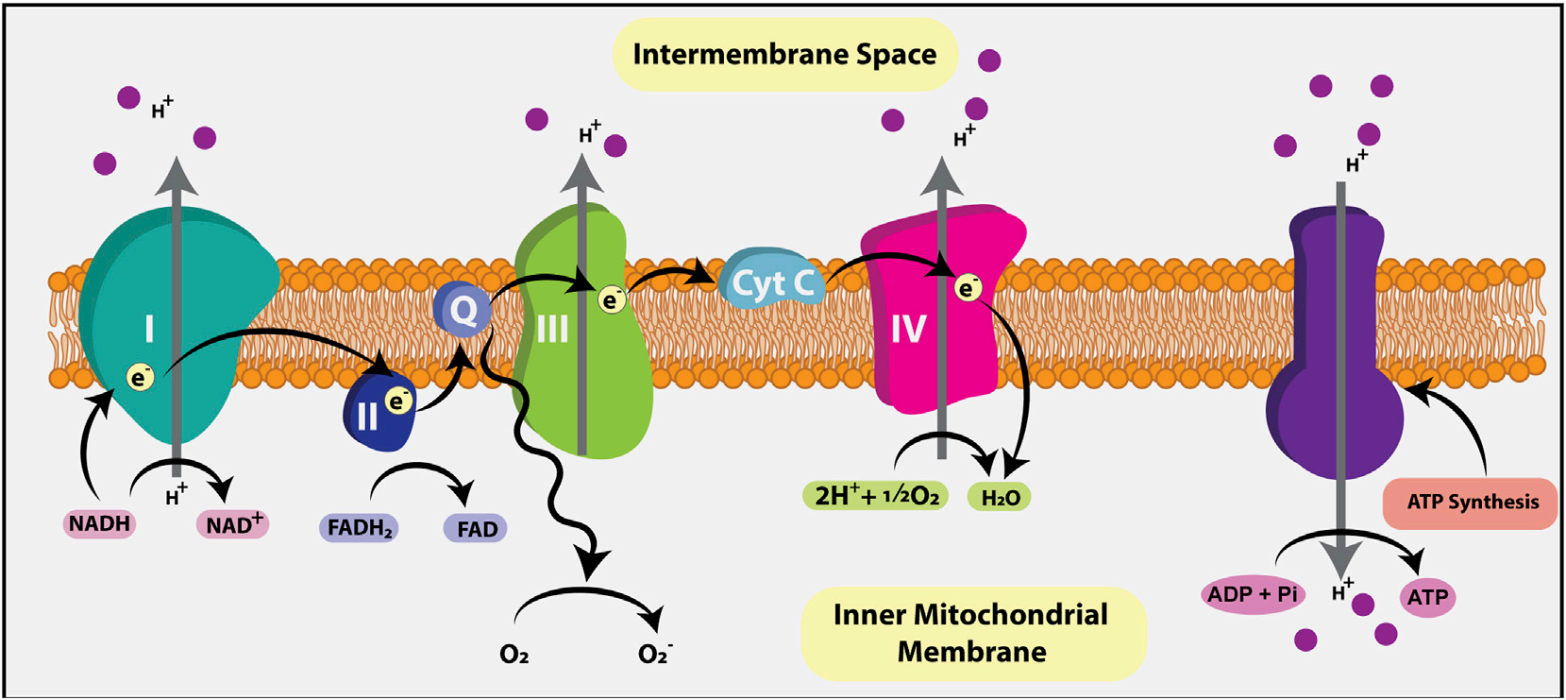
Electron transport chain (ETC). The ETC generates ATP using the reducing agents NADH and FADH2, metabolic byproducts of the TCA cycle that donate electrons to the ETC. Electrons are shuttled through four transmembrane complex proteins (I-IV) in the IMM. NADH binds to complex I while FADH2 binds to complex II, initiating electron flow. The ETC uses electron transfer to pump a proton into the intermembrane space. This voltage gradient generates a proton motive force that drives O_2_-coupled ATP generation *via* ATP synthase, termed oxidative phosphorylation (OXPHOS). OXPHOS generates ∼30–38 ATP molecules per molecule of glucose.

Normally, when O_2_ is present, pyruvate is shuttled *via* mitochondrial pyruvate carriers (MPC) into the mitochondrial matrix where it is either converted to oxaloacetate and used for gluconeogenesis, or converted to acetyl-CoA by pyruvate dehydrogenase (PDH) (Figure 2). Within the mitochondrial matrix, the acetyl group of acetyl-CoA is transferred to oxaloacetate to form citrate, which enters the tricarboxylic acid (TCA) cycle (or Krebs cycle), where it is oxidized in a series of net exergonic reactions to generate reducing equivalents (NADH, FADH_2_) that drive electron transport and establish a proton gradient across the inner mitochondrial membrane (IMM). Electrons are shuttled through a series of four transmembrane complex proteins (I-IV) in the IMM, termed the electron transport chain (ETC). The ETC uses electron transfer to pump protons into the intermembrane space (Figure 3). This voltage gradient generates a proton motive force that drives O_2_-coupled ATP generation *via* ATP synthase, termed oxidative phosphorylation (OXPHOS) (Chandel, 2014; Nolfi-Donegan et al., 2020). OXPHOS generates ∼30–38 ATP molecules per molecule of glucose and is thus more efficient at generating ATP than glycolysis.

### mTORC1 Signaling as a Central Metabolic Regulator

In healthy, glucose-rich tissues, cell growth and/or division are stimulated by several growth factors and signaling molecules, many of which bind to receptor tyrosine kinases (RTKs). Upon RTK ligand binding, multiple downstream signal transduction cascades are activated, including the phosphoinositide-3-kinase (PI3K) pathway (Figure 1). A major role of PI3K is to phosphorylate membrane phospholipids, adding a 3’ phosphate to PIP2 (phosphatidylinositol*-*4,5-bisphosphate), to form PIP3 (phosphatidylinositol*-*3,4,5-triphosphate), which is a critical second messenger. PIP3 activates PDK1 (phosphoinositide dependent kinase 1) to activate downstream effectors such as AKT, a pro-survival signal, and mTORC1 (mechanistic target of rapamycin complex 1), a critical metabolic complex comprised of the mTOR kinase, mLST8 (mammalian lethal with Sec13 protein 8) and Raptor, a regulatory protein required for mTOR kinase activity (Comer and Parent, 2007; Stambolic et al., 1998) (Figure 1). Additionally, the complex includes two inhibitory subunits, DEPTOR (DEP domain containing mTOR interacting protein), which acts in a negative feed-forward loop to control mTOR activity levels (Saxton and Sabatini, 2017), and PRAS40, which is phosphorylated by AKT and no longer able to inhibit mTOR when cells are stimulated by insulin (Sancak et al., 2007). Conversely, PTEN (phosphatase and tensin homolog) is a lipid and protein phosphatase that dephosphorylates PIP3, converting it back to PIP2, thus serving as a critical antagonistic regulator of the PI3K/AKT/mTORC1 signaling axis (Figure 1). Thus, the loss or downregulation of PTEN elevates mTORC1 signaling, which activates the translational machinery to drive protein synthesis and fuel cell growth, as described below (Magnuson et al., 2012).

In addition to the activation of mTORC1 by systemic growth factor signaling, mTORC1 is activated by intracellular nutrients and high ATP levels (Wei et al., 2019). mTORC1 thus acts as a metabolic rheostat, which induces cell growth by triggering energy consuming, anabolic processes, including: 1) cap-dependent translation of mRNAs and ribosomal RNA (rRNA) synthesis to drive protein synthesis (Valvezan and Manning, 2019); 2) increased GLUT1 expression to facilitate glucose uptake, and stimulation of glucose metabolism *via* HIF-1a-mediated upregulation of glycolysis (Valvezan and Manning, 2019); 3) mitochondrial biogenesis through increased PGC1a activity, and 4) lipid synthesis through activation of SREBP (sterol responsive element binding protein) (Carroll and Dunlop, 2017).

Two central negative regulators suppress mTORC1 activity; tuberous sclerosis 1 (TSC1) and adenosine 5′-monophosphate (AMP)-activated kinase (AMPK), the latter a central metabolic sensor for starvation cues (i.e., sensor of high AMP/ATP, ADP/ATP ratios) (Figure 1). AMPK promotes catabolic pathways to generate ATP by breaking down macromolecules (e.g., *via* autophagy, proteolysis, etc.), while inhibiting mTORC1-induced anabolic pathways that consume ATP (e.g., protein and lipid synthesis) (Purohit and Dhawan, 2019). Taken together, mTORC1 stimulation and its careful regulation by AMPK are essential for cellular metabolism to be properly maintained and balanced.

### Metabolic Organelles: Mitochondria and Lysosomes

#### Mitochondria Function as Signaling Organelles

In addition to oxidizing glycolytic substrates to generate ATP, mitochondria produce metabolic intermediates that are required for the synthesis of nucleotides, lipids and proteins that are needed to increase cellular biomass (Martínez-Reyes and Chandel, 2020). As these bioactive metabolites can control cell fate, state and/or function, mitochondria also act as signaling organelles (Martínez-Reyes and Chandel, 2020). For example, cytochrome C, which normally transfers electrons from complex III to complex IV in the electron transport chain, can also trigger programmed cell death (i.e., apoptosis) when this electron carrier leaks from inside the inner membrane space into the cytosol (Garrido et al., 2006). In addition, inefficient electron transport or reverse electron transport generates reactive oxygen species (ROS) that act as signaling molecules in homeostasis but if not maintained at manageable levels can trigger inflammation and cell death (Martínez-Reyes and Chandel, 2020). Finally, mitochondria also influence chromatin structure through the production of α-ketoglutarate (α-KG) (Figure 2). α-KG activates ten-eleven translocation (TET) DNA demethylases and histone H3K27me3 demethylases (KDM6A/6B) (Carey et al., 2015), which remove repressive methyl marks on DNA and histones, respectively, allowing chromatin to unwind and transcription factor (TF) binding to target sites (Chandel, 2014).

### Altering Mitochondrial Mass as a Response to Metabolic Need

**FIGURE 4:**
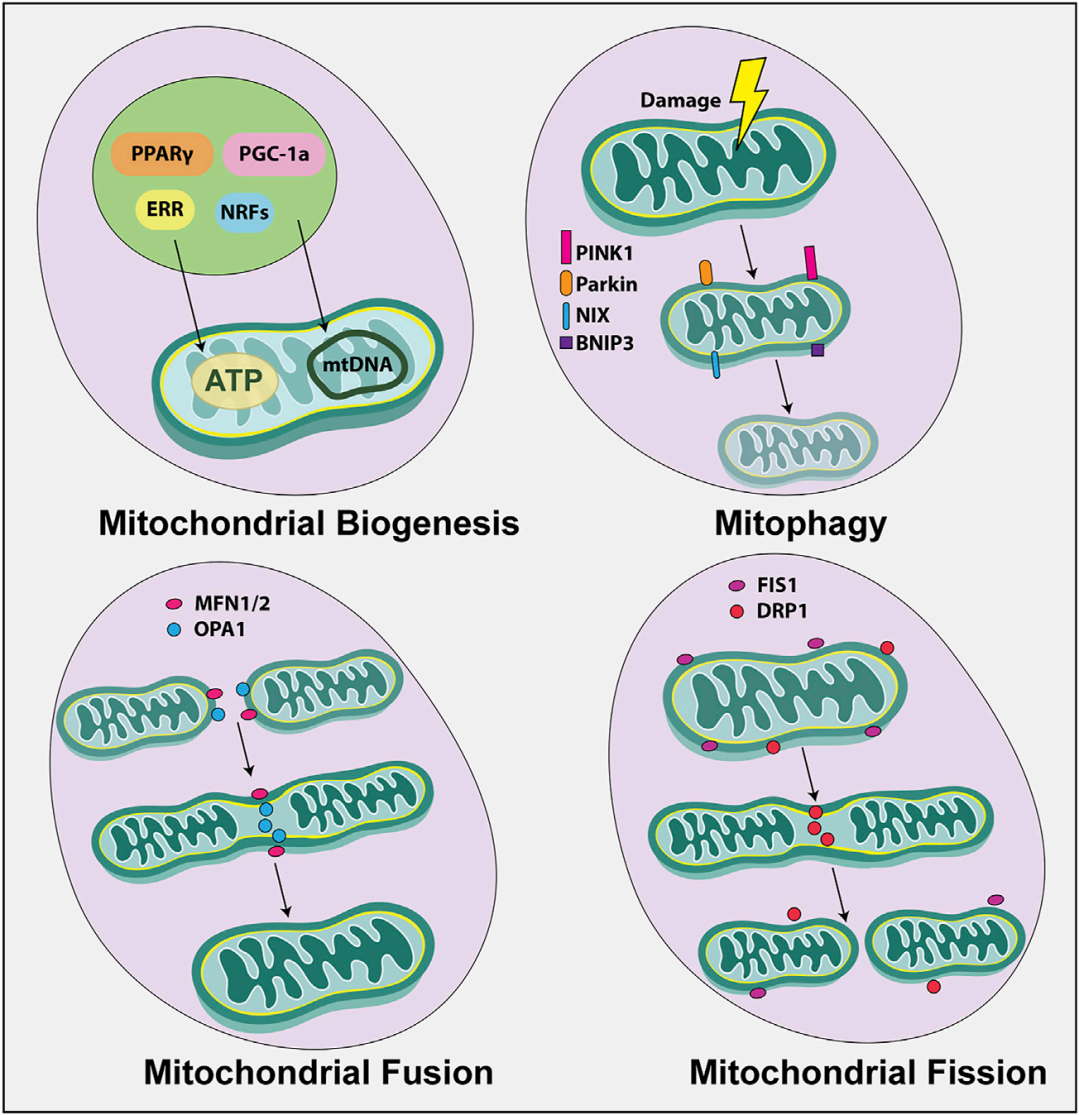
Mitochondrial dynamics. Mitochondria are dynamic structures. Mitochondrial biogenesis, or the formation of new mitochondria is triggered by transcription factors (TF), including (PPARγ) coactivator 1α (PGC-1a), estrogen-related receptor (ERR) and nuclear respiratory factors (NRFs). Mitochondrial break down, also known as mitophagy, is controlled by PTEN-induced kinase 1 (PINK), a kinase in the outer mitochondrial membrane, and Parkin, which encodes an E3-ubiquitin ligase. Mitochondria also undergo dynamic shape changes including fusion and fission. Mitochondrial fusion, which is mediated by MFN1, MFN2, and OPA1, elongates mitochondria, while mitochondrial fission or fragmentation is mediated by DRP1 and FIS1.

Finally, mitochondrial function is intimately tied to the mass of these organelles per cell and to the dynamic shape changes that they undergo (Figure 4) (Martínez-Reyes and Chandel, 2020). Mitochondrial number is balanced by mitochondrial biogenesis, or the generation of new mitochondria, and mitophagy, which is the autophagic removal of these organelles. Mitochondrial biogenesis occurs in response to energy depletion or during cell growth through the actions of several TFs, including peroxisome proliferator-activated receptor γ (PPARγ) coactivator 1α (PGC-1α), estrogen-related receptor (ERR) and nuclear respiratory factors (NRFs) (Handschin and Spiegelman, 2006). Mitophagy is regulated by two main pathways: PTEN-induced kinase 1 (PINK), a kinase in the outer mitochondrial membrane (OMM), and Parkin, which encodes an E3-ubiquitin ligase, or by an alternate NIP3-like protein X (NIX) - Bcl-2 interacting protein 3 (BNIP3) pathway (Wang et al., 2020). Finally, dynamic changes in mitochondrial shape allow cells to rapidly meet metabolic demands. Mitochondrial fusion is mediated by mitofusin (MFN) one and MFN2, OMM proteins, and Optic Atrophy 1 (OPA1), a dynamin-like GTPase, which elongates mitochondria when there is a heightened need for energy. Notably, fusion is important during the cell state transitions associated with stem cell activation in neural and hematopoietic systems (Khacho et al., 2016; Khacho et al., 2019). Conversely, mitochondrial fragmentation is mediated by dynamin-related protein 1 (DRP1), a GTPase, and fission one protein (FIS1), an OMM protein. Mitochondrial fission initiates stem cell quiescence in neural and hematopoietic systems (Khacho et al., 2016; Khacho et al., 2019). Thus, mitochondria are generally small and fragmented in quiescent neural stem cells in the brain and elongated in activated neural stem and progenitor cells.

### Lysosomes as Metabolic Sensors

Lysosomes are intracellular organelles that degrade cellular proteins by phagocytosis and other organelles by autophagy. Through this tightly regulated degradation, lysosomes serve multiple functions, including as metabolic sensors. Highlighting the importance of lysosomes as metabolic organelles, both AMPK and mTORC1 localize to the lysosome where they play a coordinated role in sensing amino acid levels as a starvation cue (Carroll and Dunlop, 2017). Leucine, arginine and glutamine are the main amino acids that at threshold levels, in nutrient-rich cells, activate mTORC1. Specifically, mTORC1 activation is controlled by Rag GTPases, which at threshold amino acid levels, serve as a docking-sites for mTORC1 on lysosomes (Sancak et al., 2010). Once on the lysosomal surface, mTORC1 is in the same compartment as Rheb, a GTP-binding protein that activates mTORC1 (Sancak et al., 2010).

Conversely, AMPK is activated in response to starvation cues, with phosphorylation by LKB1 triggering pAMPK to localize to the lysosomal membrane to downregulate mTORC1 activity (Garcia and Shaw, 2017). Upon activation, AMPK activates Ulk1, which is the main autophagy-inducing kinase that controls several autophagy (Atg)- related proteins, and can also activate mitophagy (Li and Chen, 2019). Given the central role of lysosomes as metabolic sensors, it is not surprising that lysosomal integrity is essential for cell health, with the disruption of these organelles activating several cell death pathways, including apoptosis, necroptosis, pyroptosis and ferroptosis (Zhu et al., 2020).

## The Role of Metabolism in Photoreceptor Function and Survival

### Retinal Structure and Visual Signal Processing

**FIGURE 5:**
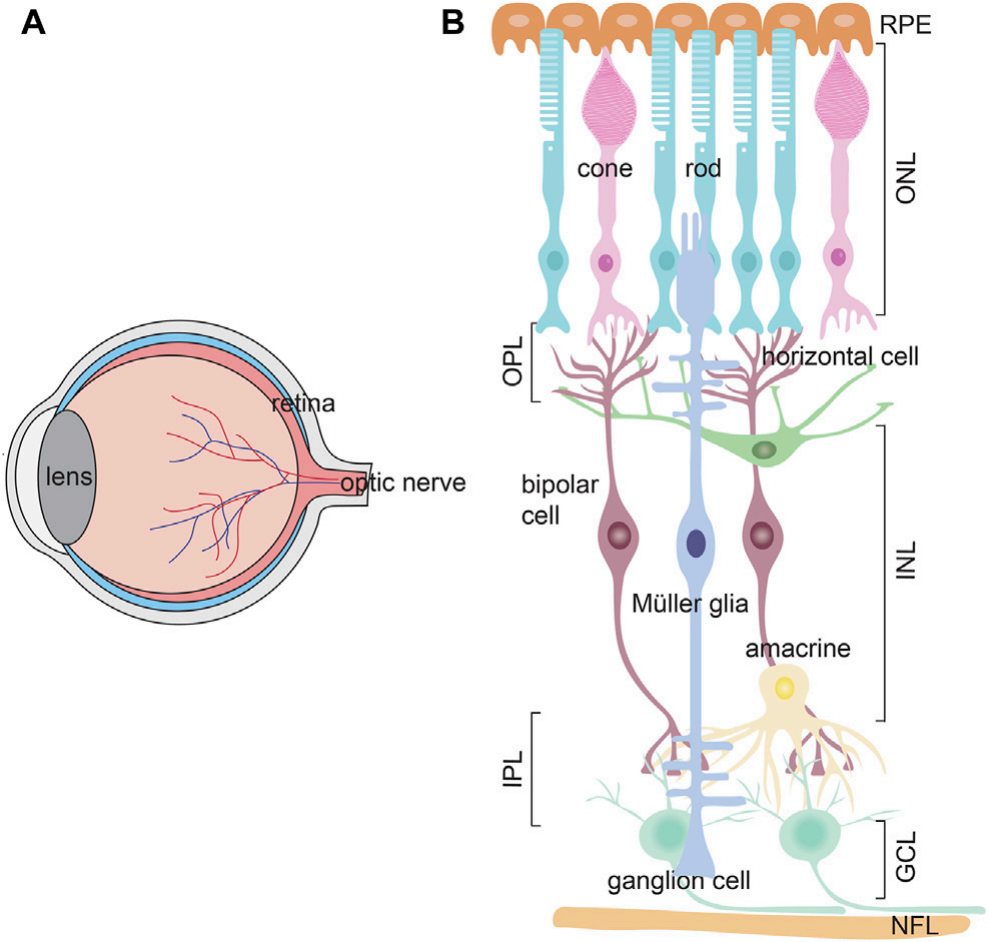
Retinal structure **(A)** Schematic of the eye, showing the lens, retina and optic nerve **(B)** Schematic of the three retinal cell layers; ganglion cell layer (GCL), inner nuclear layer (INL), and outer nuclear layer (ONL). The ONL contains rod and cone photoreceptors, which process light information. The INL contains three interneuron types–amacrine cells, bipolar cells, and horizontal cells, which modulate signals from photoreceptors, and transmit this information to ganglion cells in the GCL. Ganglion cell axons form a nerve fiber layer (NFL) and project to the brain. Müller glia (mg) are the main retinal glia with nuclei residing in the INL. There are two plexiform layers where synapses form–the outer plexiform layer (OPL) and inner plexiform layer (IPL). The apical side of the retina is covered by a retinal pigment epithelium (RPE).

The retina is a neural layer at the back of the eye that captures and processes light signals, sending this information to the brain so that animals can see (Figure 5A). There are three cellular layers in the retina; the outer nuclear (ONL), inner nuclear (INL) and ganglion cell (GCL) layers, separated by two synaptic layers, the outer plexiform layer (OPL), between the ONL and INL, and the inner plexiform layer, between the INL and GCL. The ONL contains rod and cone photoreceptors, photosensitive neurons that respond to light and transmit electrical impulses to bipolar cells in the INL. Amacrine, bipolar and horizontal cells are interneurons that reside in the INL and modulate photoreceptor signals. Ultimately, bipolar cells transmit processed visual signals to ganglion cells in the GCL, the output neurons of the retina that extend their axons to visual centers in the brain (Figure 5B). Finally, there is a single main type of glial cell–the Müller glia, which maintain structural integrity by extending processes both apically and basally to span the retina. Müller cells also have growth supportive and injury-responding roles, described further below, and act as optical fibers that orient light towards photoreceptors to minimize light scatter (Franze et al., 2007).

The cells in the three retinal layers intimately coordinate with each other so that visual information can be sent to the brain for processing *via* the optic nerve (Baden et al., 2020). Briefly, the retinal visual cycle begins with rod and cone photoreceptors, which carry out phototransduction. The two photoreceptor types differ in the type of light that they recognize and process—rods sense dim light and mediate scotopic, night vision, while cones mediate color and high acuity photopic vision. In their outer segments, rods contain rhodopsin, a G-protein coupled receptor (GPCR) that is much more sensitive to light than the opsins in the cone outer segments, but rhodopsin rapidly photo-bleaches. Mammalian retinas typically have three cone types distinguished by the peak absorption wavelength of the opsin they express. Long wavelength (L)-cones express the opsin OPN1LW and detect red light (∼564 nm) in the visible spectrum, medium wavelength (M)-cones express OPN1MW to capture green light (∼534 nm), and short wavelength (S)-cones express OPN1SW to capture blue light (∼420 nm). Rodent retinas only contain M- and S-cones, whereas human retinas have all three types of cones (Nadal-Nicolás et al., 2020).

**FIGURE 6:**
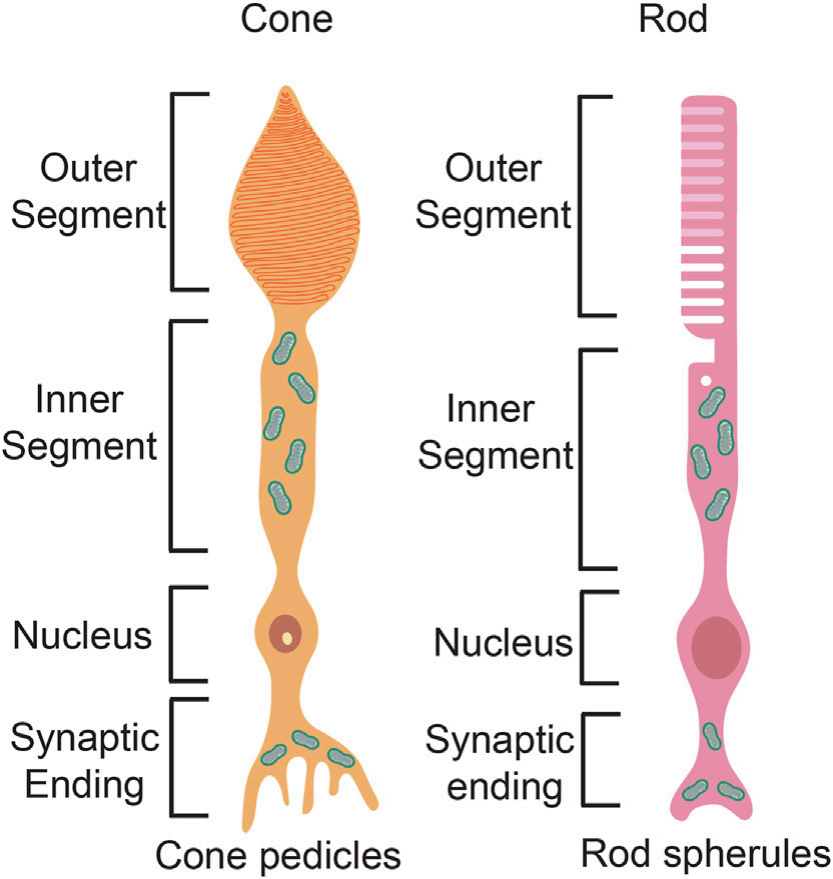
Photoreceptor structure. Schematic representation of a rod photoreceptor and a cone photoreceptor, highlighting differences in the basal axon and the apical processes.

Rod and cone photoreceptors differ in their cell morphologies. Both photoreceptor types extend a basal axon that terminates in the outer plexiform layer as a spherule in rods and a pedicle in cones (Figure 6). Rods and cones also have the same apical outer processes, including an outer segment (OS), where phototransduction occurs, which is connected *via* a cilium to an inner segment (IS), where mitochondria accumulate and ATP is generated (Figure 6). Critical differences between rods and cones occur in the OS membranes. In rods, the OS membranes have a rod-like structure, and are folded into discontinuous stacks of discs that are efficient at trapping light. Conversely, in cones, the disc membranes are continuous with the plasma membrane and form a cone-like shape. Both rod and cone discs are constantly replaced by the formation of new discs, while old discs undergo RPE-mediated phagocytosis as they undergo photo-oxidative damage over time (Kocaoglu et al., 2016).

### Nutrient and Oxygen Supply in the Mature Retina

**FIGURE 7:**
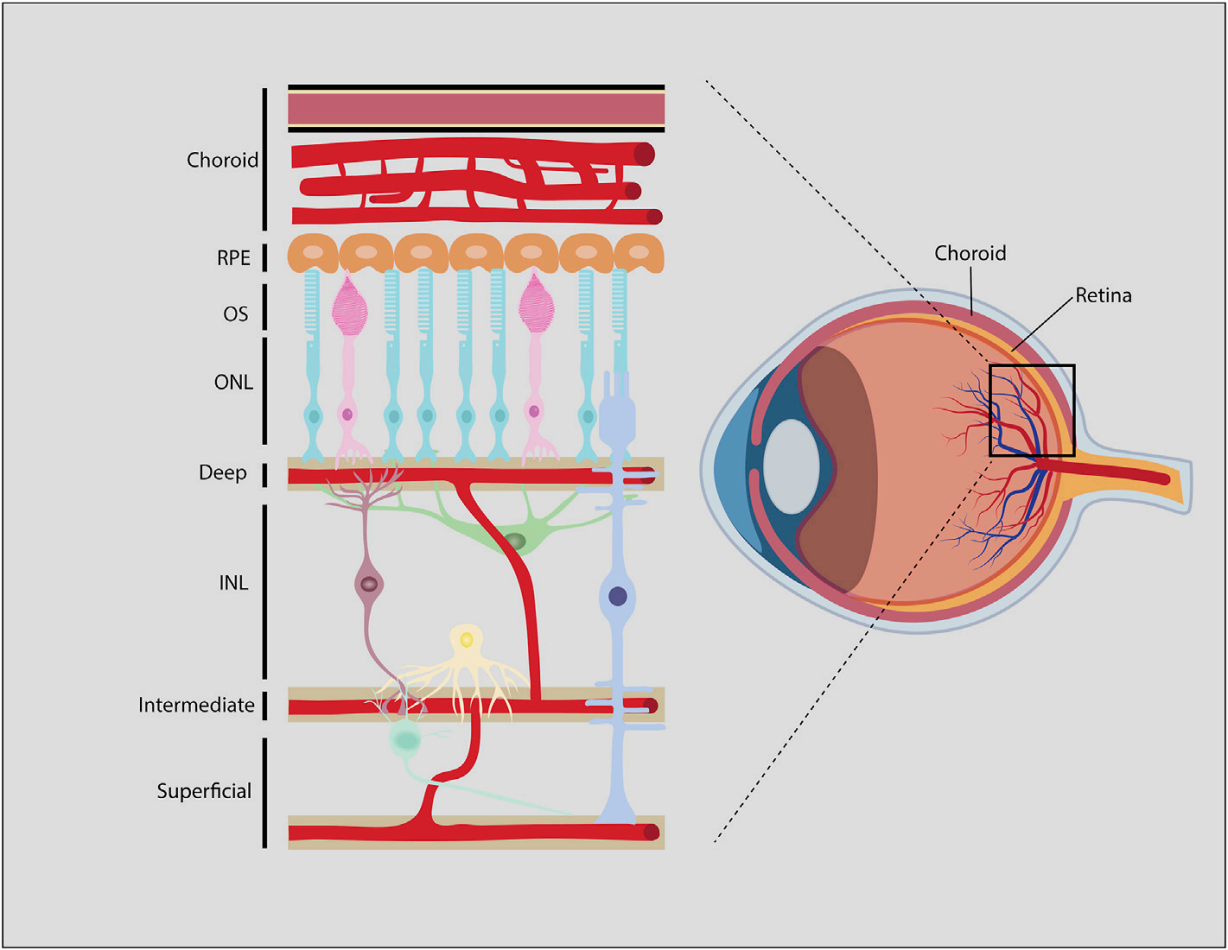
Blood-retinal-barrier (BRB). Generalized schematic of the mammalian BRB, showing retinal vessels, some species like zebrafish, chicken and rabbit may have different vascular architecture. Endothelial cells and associated pericytes that line the deep, intermediate, and superficial vascular plexi make up the inner BRB (iBRB). The RPE is part of the outer BRB (oBRB) that prevents blood-borne factors from entering the retina from the choroidal vessels.

The retina is one of the highest energy-demanding organs in the body, consuming oxygen at higher rates even than the brain (Hurley et al., 2015). Photoreceptors are the main energy-consuming retinal cells, and consume ATP both in light and darkness (Hurley et al., 2015; Okawa et al., 2008). Each retinal layer receives O_2_ and nutrients, predominantly glucose, through the ocular vasculature (Figure 7). The ONL, where photoreceptors reside, is itself avascular, with choroidal vessels overlying the ONL providing the main vascular supply of nutrients to photoreceptors. In contrast, inner retinal neurons receive nutrients and oxygen from three inner retinal vascular plexuses; a superficial plexus that emerges directly from the central retinal artery perfuses the GCL, while two additional plexuses, labeled intermediate and deep, infiltrate the INL. These three plexuses are connected *via* vertical anastomoses and are evident in human, non-human primate and rodent retinas (Selvam et al., 2018).

The retinal vasculature is distinct from other capillary networks in the body. Similar to the blood-brain-barrier (BBB) (Campbell and Humphries, 2012), the blood-retinal-barrier (BRB) includes tight junctions between endothelial cells and pericytes that surround the retinal capillaries (Díaz-Coránguez et al., 2017). The outer BRB is a barrier between the choroidal vessels and the retinal pigment epithelium (RPE), which is a pigmented single cell layer that overlies the ONL and underlies the choroidal vasculature (Díaz-Coránguez et al., 2017). The inner BRB is a barrier between the deep, intermediate and superficial vascular plexuses and the retinal tissue that they innervate. Together, the outer and inner BRB allows the passage of nutrients and oxygen that circulate in the choroidal and inner retinal vessels, but prevents the entry of antibodies, immune cells and most therapeutics that are administered systemically (Antonetti et al., 1999).

### Retinal Pigment Epithelial Protection and Photoreceptor Survival–Role of Metabolism

#### Retinal Pigment Epithelium and Glucose Supply to Photoreceptors

The RPE, which is sandwiched between the photoreceptor layer and choroid, plays a critical role in maintaining metabolic homeostasis, supplying up to 60% of the overall glucose consumed by the retina, as well as removing waste products. Substance exchange is facilitated by microvilli on the apical surface of RPE cells, which increases their cellular surface area, while absorption of nutrients on the basal side is maximized by invaginations (Boulton and Dayhaw-Barker, 2001; Bonilha et al., 2006). Specialized transport proteins on the apical and basal cellular surfaces of the RPE also control ion, nutrient and metabolite transfer between the choroid and retina (Sugasawa et al., 1994). The importance of glucose transport by the RPE for retinal health was evidenced by the tissue-specific conditional deletion of *Glut1* in murine RPE cells, which led to a shortening of photoreceptor outer segments, and ultimately photoreceptor cell death (Swarup et al., 2019). In response to stress, RPE cells increase glycolytic metabolism and reduce the transport of glucose to photoreceptors, which die from starvation (Pan et al., 2021). In addition, choroidal and retinal blood flow are reduced in patients with AMD, a triggering stimulus for photoreceptor degeneration that is exacerbated by obesity, smoking and other environmental triggers that compromise blood vessel health (O'Leary and Campbell, 2021). Mechanistically, these different triggers all induce oxidative stress, which leads to RPE cells stockpiling glucose and fat such that they transport less nutrients to the retina, triggering photoreceptor degeneration (Kurihara et al., 2016).

#### Retinal Pigment Epithelial Defects and Altered Lipid Metabolism in Age-Related Macular Degeneration

In AMD, RPE cells undergo regional or geographic atrophy in the central cone-rich macula, a degenerative process that is associated with the accumulation of lipid-rich deposits such as lipofuscin within and drusen outside the RPE (Inana et al., 2018). Aberrant lipid metabolism is included among the large number of environmental and genetic factors that increase AMD risk. Indeed, a major component of drusen in AMD patients is Apolipoprotein E (APOE), a carrier for lipids and cholesterol that is aberrantly deposited by the RPE in AMD patients (van Leeuwen et al., 2018). APOE has different allele haplotypes, with the E2 allele moderately increasing AMD risk, while the E4 allele surprisingly displays some protection against AMD, even though it is a risk factor for Alzheimer’s disease (Anderson et al., 2001). Additional AMD-associated alleles are found in other genes related to lipid metabolism, such as *APOC2*, *ABCA1* and *CETP*, linking disorders of lipoprotein metabolism to disease (Deng et al., 2022). Moreover, high levels of high-density lipoprotein cholesterol confer increased risk to AMD and is associated with the accumulation of lipid peroxidation end products in lipofuscin (van Leeuwen et al., 2018). This increase in lipid oxidation is related to elevated levels of ROS in AMD, as described further below. In addition, as drusen accumulates, it is thought to form a lipid wall that reduces glucose transport to photoreceptors (Curcio, 2018). Given these linkages to altered lipid metabolism, it is not surprising that an association between metabolic syndrome and AMD has been reported (Ghaem Maralani et al., 2015). Preventative strategies based on diet have thus begun to be tested, with dietary omega-3 fatty acids and antioxidants showing some evidence of protection against AMD (van Leeuwen et al., 2018).

#### Mitochondrial Role in Retinal Pigment Epithelial Function

As highly metabolic cells, RPE cells have high energy demands that are met by producing large numbers of metabolic organelles, including mitochondria (Boulton and Dayhaw-Barker, 2001), the dysfunction of which is implicated in AMD (Terluk et al., 2015; Fisher and Ferrington, 2018; Brown et al., 2019). Indeed, in the RPE of patients with AMD, there is a reduction in mitochondrial mass (Feher et al., 2006), lower levels of electron transport chain proteins (Nordgaard et al., 2008), and mitochondrial DNA (mtDNA) damage (Karunadharma et al., 2010; Lin et al., 2011). As a consequence of mitochondrial insufficiency, ROS accumulate and respiration is defective in the RPE of patients with AMD (King et al., 2004). Supporting the importance of mitochondria for RPE function, an RPE-specific conditional knock-out (cKO) of mitochondrial transcription factor A (*Tfam*), which transcribes mitochondrial genes, leads to RPE hypertrophy, RPE dedifferentiation and progressive photoreceptor degeneration (Zhao et al., 2011). In RPE-specific *Tfam* cKOs, electron transport chain function is reduced even though mitochondrial size is increased, which in an apparent attempt to compensate for reduced energy production. In addition, RPE mTOR signaling is elevated in *Tfam* cKOs, and there is an associated increase in glycolysis (Zhao et al., 2011). This metabolic shift is central to the pathogenic features observed, as treating *Tfam* cKO mice with rapamycin, an mTOR inhibitor, suppresses RPE dedifferentiation and preserves photoreceptor health (Zhao et al., 2011). Thus, consistent with observations made in other tissues, suppressing mTOR signaling preserves retinal function during aging, an interesting feature given that suppression of this pathway is also associated with increased lifespan (Johnson et al., 2013).

Mitochondria are dynamic structures, with fission, or the division of these organelles to form new mitochondria, linked to shorter lifespans in *S. cerevisiae* (budding yeast) (Scheckhuber et al., 2007) and *C. elegans* (Kim et al., 2011), as well as premature aging and neurodegenerative diseases in mammalian species (Liu et al., 2020). To assess the role of mitochondrial fission in RPE function, an RPE-specific *Pgam5* cKO, was generated (Yu et al., 2020). *Pgam5* encodes a mitochondrial phosphatase that dephosphorylates DRP1 to promote fission, the loss of which leads to mitochondrial hyperfusion, which in turn reduces mitochondrial turnover and subsequently leads to elevated mTOR signaling, ATP and ROS production. As a consequence, in RPE-specific *Pgam5* cKOs, RPE cells enter cellular senescence (Yu et al., 2020), or permanent growth arrest, a common feature of aging (Tuttle et al., 2020). Notably, the senescent phenotype can be reverted by the expression of *Drp1* in which the phospho-acceptor sites recognized by PGAM5 are mutated (Yu et al., 2020). While this study did not investigate photoreceptor degeneration, it does provide important information on the role of mitochondrial morphology in RPE cell health. Consistent with a potential role in retinal neurodegeneration, *Pgam5* loss-of-function mutants display a Parkinson’s disease-like movement disorder that is associated with the appearance of dysfunctional mitochondria and cellular senescence (Lu et al., 2014). Moreover, senescent cells accumulate in many age-related diseases, and their elimination with senolytic drugs is a treatment strategy to help restore tissue homeostasis in neurodegenerative diseases (Baar et al., 2017; Childs et al., 2017). There is thus emerging support for a link between the deregulation of mitochondrial dynamics, the acquisition of a senescent cellular phenotype, and neurodegeneration.

#### Oxidative Stress in the Retinal Pigment Epithelium in AMD

The RPE plays a central role in protecting the retina from oxidative damage by the production of antioxidants, such as superoxide dismutase and glutathione. Treatment strategies that protect against oxidative stress have been tested in animal models of AMD. Strikingly, metformin, which stimulates AMPK signaling, mitochondrial biogenesis and ATP production *via* glycolysis, confers resistance to oxidative stress (e.g., sodium iodate-induced injury) and survival of RPE cells and photoreceptors (Xu et al., 2018). However, blocking oxidative stress alone, for example by delivering *Sod2* using an AAV to RPE-specific *Sod2* cKO mice, does not effectively prevent the appearance of AMD-like symptoms, especially once photoreceptor degeneration has progressed (Biswal et al., 2017). It has thus been proposed that metformin acts in other ways, such as its ability to reduce gluconeogenesis, to confer protection in animal models of AMD. Regardless of its mode of action, metformin is now being tested in three retinal neurodegenerative diseases in clinical trials (clinicaltrials.gov), including in patients with AMD (NCT02684578). However, metformin is not the panacea, as its’ protective effects appear to be restricted to metabolic disorders associated with increased oxidative stress. Indeed, rod photoreceptor degeneration is instead accelerated when metformin is applied in a protein-folding disorder of the retina (Athanasiou et al., 2017). Understanding disease etiology and metformin function is thus critical to the future application of this drug to treating patients with retinal degenerative diseases.

### Photoreceptor Metabolism and Links to Cell Dysfunction, Cell Death and Retinal Disease

#### Metabolic Demand of Photoreceptors

Photoreceptors (i.e., rods and cones) are highly metabolically active, consuming most of the energy supply to the retina in the form of ATP to sustain phototransduction (Narayan et al., 2017). The main energy requirement in the outer segment is the need to keep cGMP-gated, cation-selective ion channels in an open state so that cations such as Na^+^ and Ca^2+^ can enter and continuously depolarize the membrane (Okawa et al., 2008). Photoreceptors differ from most other CNS neurons in that they have a depolarized membrane potential of −40 mV when not stimulated (i.e., in darkness), and become hyperpolarized in response to progressive illumination, saturating at −65 mV (Pan et al., 2021). In the dark, high cGMP levels in the outer segments allow Na^+^ and Ca^2+^ to enter and depolarize the membrane (Okawa et al., 2008). Upon photo-stimulation, cGMP is hydrolyzed, reducing the need for ATP to open cGMP-gated channels and preventing Na^+^ and Ca^2+^ from entering the outer segments such that photoreceptor cell membranes become hyperpolarized, leading to a lower rate of neurotransmitter release into the synapse (Okawa et al., 2008). Notably, as cones are less light sensitive and more often in an inactive, depolarized state compared to rods, cones are the more energetically demanding cell type (Nikonov et al., 2006; Ingram et al., 2020).

During phototransduction, light photons provide the energy that drives a configurational change of 11-cis-retinal, an opsin-linked chromophore, to all-trans-retinal, which activates transducin, a G protein that is then activated by switching from GDP to GTP binding. The signal is amplified as one light photon activates around 400 transducin molecules (Heck and Hofmann, 2001). Transducins then activate phosphodiesterase-6, which hydrolyzes cGMP intracellularly to form GMP. The resulting reduction of cGMP levels leads to the closure of ion channels causing further hyperpolarization of the membrane.

#### Glycolysis as the Main Bioenergetic Pathway Used by Photoreceptors

While mature CNS neurons generally use OXPHOS to generate ATP, photoreceptors are an exception, with ∼80–90% of their energy production coming from aerobic glycolysis (Wang et al., 1997a; Petit et al., 2018). The requirement for aerobic glycolysis is especially important for rod photoreceptor function, with rod function perturbed more than cone function when hexokinase 2 (*Hk2*), a gene encoding an enzyme that is a gatekeeper of aerobic glycolysis, is knocked-out specifically in photoreceptors (Petit et al., 2018). Notably, earlier studies in the cat eye similarly showed a larger effect of reduced glucose levels on rod versus cone function, with cones only affected when glucose levels were sharply reduced (Macaluso et al., 1992).

Photoreceptors must balance the need to produce large amounts of ATP through catabolic pathways with a high anabolic demand that comes from the daily requirement to replace outer segments. During “housekeeping glycolysis”, glucose is broken down to pyruvate in a multi-enzymatic cascade, the last step involving pyruvate kinase (PK), which exists in two isoforms–PKM1, which is constitutively active, and PKM2, which is activated by tetramerization (Pan et al., 2021). A recent study found that PKM2’s role in generating pyruvate is essential for outer segment maintenance (Chinchore et al., 2017). Pyruvate is then reduced to lactate *via* lactate dehydrogenase (Ldha), the knockdown of which also inhibits outer segment biogenesis (Chinchore et al., 2017). Similar reductions in outer segment length were observed upon overexpression of *Tigar* (TP53-induced glycolysis and apoptosis regulator), which functionally mimics the knockdown of 6-phosphofructo-1-kinase (Pfk1), an enzyme that commits the use of glucose metabolites to glycolytic flux (Chinchore et al., 2017). Thus, glycolysis is required to meet the energy demands of phototransduction and for the anabolic process of outer segment maintenance.

#### Rod-Derived Cone Viability Factor Regulates Glucose Uptake and Photoreceptor Survival

Rod photoreceptors, which outnumber cone photoreceptors 20:1 in humans (Massey et al., 2006) and 35:1 in mice (Jeon et al., 1998), are also essential for cone survival, acting in part through the secretion of rod-derived cone viability factor (RdCVF). RdCVF is a thioredoxin-like factor that binds the Basigin-1 (Bsg1) receptor on cones (Byrne et al., 2015). Alternative splicing of Nucleoredoxin-like 1 (*Nrxnl1*) produces full-length and truncated RdCVF isoforms in rods, with the truncated form providing neurotrophic support to cones, and the longer form controlling oxidative signaling (Byrne et al., 2015). Consequently, there are defects in both rod and cone function in *Nrxnl1* KOs, as well as progressive cone degeneration. Mechanistically, RdCVF forms a complex with Bsg1 and Glut1 to increase glucose uptake (Yang et al., 2009). Thus, when RdCVF is administered to *Pde6b* ^rd1^ mice, a model of RP, aerobic glycolysis and ATP production are elevated and photoreceptor degeneration is prevented (Aït-Ali et al., 2015). The neuroprotective effects of RdCVF are thus achieved at least in part by stimulating glucose metabolism (Aït-Ali et al., 2015).

Given the high metabolic demand of photoreceptors, it is not surprising that energy failure stemming from defects in metabolism is an underlying factor in many neurodegenerative disorders of the retina (Narayan et al., 2017; Pan et al., 2021). Indeed, mutations in glycolytic genes, such as *HK1* and *HKDC1*, are associated with RP in patients (Wang et al., 2014; Zhang et al., 2018; Pan et al., 2021). Similarly, a photoreceptor-specific *Hk2* cKO in mouse, which blocks aerobic glycolysis, leads to a diminished capacity of rods and cones to respond to nutrient stress, even though they generate more mitochondria and efficiently use OXPHOS as an adaptive metabolic response (Petit et al., 2018).

#### Nutrient and Metabolite “Sharing” Between Cell Types Creates a Metabolic Ecosystem

The retina can be viewed as a metabolic ecosystem in which cells “share” nutrients and metabolites to sustain their function and health (Kanow et al., 2017; Jaroszynska et al., 2021). Indeed, in the outer retina, rod and cone photoreceptors form an integrated network with the RPE, Müller glia, and with each other, and there is growing evidence that disruption of this ecosystem leads to metabolic failure, photoreceptor death and vision loss (Jaroszynska et al., 2021). For instance, rods and Müller glia secrete large amounts of lactate as a glycolytic byproduct that is taken up by other cells as a metabolic fuel source, including by the RPE (Winkler et al., 2004; Kanow et al., 2017; Jaroszynska et al., 2021). By taking up lactate, which is a carbon source for OXPHOS, glycolysis is suppressed in RPE cells, such that less glucose is used up and more can be transferred to photoreceptors (Kanow et al., 2017). It is thus not surprising that defects in photoreceptor metabolism have non cell autonomous effects on other cells in the retina. For instance, as described in more detail in the next section, rod loss disrupts nutrient support from the RPE to cones, which die due to “starvation”, or reduced mTORC1 signaling (Punzo et al., 2009; Venkatesh et al., 2015). In this regard, it is interesting that in transgenic mice carrying a P23H RHO mutation, a model of RP, the reduced expression of metabolic genes in photoreceptors is mirrored by an upregulation of these same genes in Müller glia in an apparent attempt to compensate (Tomita et al., 2021). Thus, the impact of alterations in cell metabolism cannot be considered in isolation for a single cell type.

#### Role of mTOR and Upstream Signal Transduction Molecules in Photoreceptor Health

In RP, rod photoreceptors degenerate first, followed by cone photoreceptors, demonstrating the essential role that rods play in sustaining cone cell health, including in supporting the flow of nutrients from the RPE (Punzo et al., 2009; Venkatesh et al., 2015). Accordingly, in transgenic mouse models of RP, there is an association between photoreceptor cell death and reduced signaling by the metabolic regulators, AKT, PI3K, and mTOR (Jomary et al., 2006; Punzo et al., 2009; Ivanovic et al., 2011; Venkatesh et al., 2015). Highlighting the functional significance of these findings, in cone-specific Rptor;Rictor double cKOs (ablating mTORC1 and mTORC2 activity), there is an age-related decline in cone function and outer segment abnormalities, albeit without impacting cone survival (Ma et al., 2015). Conversely, insulin-induced metabolic reprogramming in a *Pde6b* ^–/–^ mouse model of RP increases mTORC1 signaling and improves cone survival (Punzo et al., 2009). Similarly, elevating mTORC1 signaling in cone photoreceptors by crossing *Pten* cKO or *Tsc1* cKO alleles into *Pde6b* ^*rd1*^ mice (Venkatesh et al., 2015), or crossing a rod specific *Tsc1* cKO allele into *Pde6b* ^*H620Q/H620Q*^ RP mutant mice (Zhang et al., 2016), prevents cone and rod degeneration, respectively. Activation of mTORC1 signaling in these models is associated with increased expression of genes driving glucose transport (Glut1), glycolysis (Hif1a, Hk2, PK-M2) and glucose-6-phosphate dehydrogenase (G6PD), required to make NADPH from glucose in the pentose phosphate pathway (Venkatesh et al., 2015). Moreover, CASP2-dependent cell death is activated by low NADPH levels, the removal of which in *Pde6b* ^*rd1*^;*Casp2* ^*−/−*^ mice improved cone survival, demonstrating how reduced glucose metabolism may contribute to cone cell death (Venkatesh et al., 2015).

Despite these striking findings suggesting that high mTORC1 is neuroprotective in the retina, the path moving forward is not clear, as a separate study found that elevating mTORC1 in cones leads to late-stage AMD-like symptoms, including the presence of drusen-like deposits, accumulation of lipoproteins and RPE atrophy (Cheng et al., 2020). In *Tsc1* cKOs, mTORC1 signaling was elevated in photoreceptors, which displayed hallmarks of senescence (p16Ink4a and p21 expression, senescence associated-β-galactosidase activity) and photoreceptor death (Rao et al., 2021). Similarly, mTORC1 signaling is elevated in a chemical model of RP, leading to photoreceptor degeneration, and rapamycin could slow down this degenerative response (Zhao et al., 2022). Additionally, metformin, which has neuroprotective effects in both chemical and genetic models of retinal neurodegeneration (Xu et al., 2018), activates AMPK to block mTORC1 signaling (Reh, 2016). Additional studies are thus required to tease apart how both the elevation and reduction of mTORC1 signaling can be neuroprotective in different models of retinal disease. A likely possibility is that any change to mTORC1 activity deregulates the capacity of cells to respond to nutrient supply through the pleiotropic effects of this signaling complex (Venkatesh et al., 2015). An alternative approach may be to target glycolysis directly, which would avoid the multiple effects of manipulating mTOR signaling. In this regard, it is important to highlight a recent study in which a mutated form of Arrestin1 was shown to increase glycolysis and lactate production by disinhibiting Eno1, leading to improved photoreceptor survival in *Rho* ^*P23H/+*^ mice (Nelson et al., 2022).

#### Assessing Mitochondrial Function in Photoreceptors

Mitochondria are abundant in the inner segments of both rods and cones (Figure 6), raising the question of their main function(s). Photoreceptors convert 80% of glucose to lactate *via* glycolysis which produces ATP and metabolic intermediates for outer segment biogenesis (Wang et al., 1997a; Wang et al., 1997b). However, OXPHOS also contributes to energy production in the inner segments, generating ATP to fuel cGMP-gated channels to repolarize photoreceptor membranes in the dark (Okawa et al., 2008). Accordingly, mutations in TCA cycle genes, such as IDH3B have been identified in patients with nonsyndromic RP (Fattal-Valevski et al., 2017; Pierrache et al., 2017), highlighting the importance of mitochondria in photoreceptor survival. Notably, given that 80% of glucose is metabolized by glycolysis, other carbon sources, such as lipids, including the long-chain fatty acid palmitate, are thought to fuel the TCA cycle in photoreceptor inner segments (Joyal et al., 2016). Finally, photoreceptor mitochondria in the inner segment also regulate Ca^2+^ ion homeostasis, regulating levels of this important cation to regulate membrane depolarization and neurotransmission (Jaroszynska et al., 2021).

#### Role of the Lysosome in Photoreceptor Health

Lysosomes play a critical role in sustaining photoreceptor health and function, beginning with their critical role in phototransduction through their degradation of photoreceptor opsins, including rhodopsin, which must be continually replaced (Santo and Conte, 2021). In addition, circadian cycles of lysosome-mediated autophagy are observed in the rod inner segment following the peak of disc shedding, which is triggered by the constant exposure of photoreceptors to light and subsequent oxidative stress (Santo and Conte, 2021; Reme´ et al., 1986). Given the important role that lysosomes play in protein and organelle clearing and metabolic sensing, it is not surprising that several lysosomal storage disorders are associated with retinal dystrophies (Intartaglia et al., 2020) and other neurodegenerative diseases (Zhu et al., 2020). Indeed, autophagy flux is elevated in *RHO* ^*P23H*^ mice, a model of RP, and the genetic or pharmacological inhibition of autophagy promotes photoreceptor function and survival in these mice (Yao J. et al., 2018). Similarly, autophagy is increased in response to light damage in the retina, and autophagy inhibitors were shown to reduce light-induced photoreceptor cell death (Kunchithapautham and Rohrer, 2007). Conversely, even though autophagy is reduced in Rd10 mice, activating autophagy with rapamycin, an mTOR inhibitor, accelerates photoreceptor cell death (Rodríguez-Muela et al., 2015). However, the field is not without controversy, as other studies have reported that experimental induction of autophagy improves photoreceptor cell survival. Indeed, metformin activates AMPK and its protective effects on photoreceptor survival in Rd10 mice, a model of RP, have at least in part been attributed to the activation of lysosome-mediated autophagy (Xu et al., 2018). In addition, an increase in autophagy is seen in a rat model of retinal detachment, and inhibiting autophagy accelerates photoreceptor cell death (Besirli et al., 2011; Santo and Conte, 2021). Despite these controversies, which may reflect differences in model systems and assays used, there is a clear link between lysosomal dysfunction and neurodegenerative disorders of the retina. While outside the scope of this review, notable examples include Batten Disease, which is a lysosomal storage disorder associated with mutations in CLN3 that result in juvenile onset vision loss due to the death of retinal interneurons (Johnson et al., 2019). Strikingly, it was recently demonstrated that AAV-mediated rescue of CLN3 expression improves bipolar cell survival and sustains vision in a mouse model, providing hope for new therapies in the future (Kleine Holthaus et al., 2020).

### Future Directions

#### Deciphering Metabolic Changes in Retinal Degenerative Diseases

So far, many of the studies linking reduced energy metabolism to retinal degeneration have either been correlative (i.e., mutations in glycolytic genes) or examined the effects of altering mTORC1 signaling in animal models. A question that remains is whether metabolic rate reductions are indeed observed in the retina during neurodegenerative disease. Interestingly, Fluoro-Deoxy-Glucose Positron Emission Tomography (FDG-PET) has been applied to individuals with Alzheimer’s disease, revealing reduced rate of glucose metabolism that can distinguish these patients from those with other types of dementia (e.g., frontotemporal dementia, Lewy body disease) (Marcus et al., 2014). FDG-PET has also been used to examine glucose metabolism in the retina and FDG uptake is observed in Müller glia (Poitry-Yamate et al., 2013). In zebrafish models, live imaging of 2-NBDG uptake, a fluorescent glucose analog, has been used in screens for drugs that alter glucose flux in a model of diabetes (Jaroszynska et al., 2021). Future experiments could investigate whether similar metabolic abnormalities can be used for early diagnosis and to distinguish patterns of degeneration in AMD and RP.

#### Identifying the Contribution of Mitochondrial-Dependent Apoptotic Cell Death to Retinal Degenerative Diseases

Neurons have preset genetic programs in place that ensure they are maintained for the lifetime of an animal (Lin et al., 2020). A recent study revealed that this neuronal survival program occurs at the level of the mitochondria with post-transcriptional regulation of the pro-apoptotic protein BAK1 attenuating apoptosis in the brain (Lin et al., 2020). Whether a similar survival mechanism occurs in retinal neurons, and furthermore, whether mitochondrial-dependent cell death pathways are perturbed in retinal neurodegenerative disease remains to be determined. However, like the brain, BAK1 expression is normally excluded from the retina, likely through the same nonsense medicated decay (NMD) pathway (Lin et al., 2020). Moreover, as highlighted above, CASP2-dependent cell death is activated by low NADPH levels, which is found in *Pde6b* ^*rd1*^ mice and associated with retinal degeneration (Venkatesh et al., 2015). In this regard, it is of interest that deleting *Nmnat1* (NAD^+^ synthase nicotinamide mononucleotide adenylyltransferase-1), which regulates the formation of NAD^+^ to shuttle electrons, in retinal progenitor cells created with a *Six3-Cre* driver leads to defects in photoreceptor maturation and survival, with multiple cell death pathways activated (i.e., apoptosis, pyroptosis, necroptosis) (Sokolov et al., 2021). Interestingly, there is a striking disruption of multiple retinal metabolites (39/112 metabolites de-regulated, as determined by LC-MS/MS), which taken together reveal defects in central carbon metabolism, including glycolytic flux (Sokolov et al., 2021). The link between these metabolic disturbances and each of the cell death pathways remains to be determined. This question is of importance as multiple mutations in NMNAT1 have been identified in patients with Leber congenital Amaurosis (LCA), another neurodegenerative disease targeting photoreceptors (Chiang et al., 2012; Falk et al., 2012; Koenekoop et al., 2012; Perrault et al., 2012).

#### Assessing Mitochondrial Fission and Fusion Dynamics in Healthy Versus Degenerating Photoreceptors

Mitochondrial fission, or fragmentation, is associated with photoreceptor degeneration in a model of retinal detachment (She et al., 2018). This degeneration occurs in response to DRP1 activation, which induces mitochondrial fission and apoptosis (She et al., 2018). In the brain, DRP1 was found to be necessary for engulfment of fragmented mitochondria in autophagic vesicles (Goldsmith et al., 2022). Interestingly, mutations of OPA1, which result in a loss of inner mitochondrial membrane fusion, lead to an autosomal dominant optic atrophy (from which OPA1 derives its name) and childhood blindness. Moreover, when an OPA1-like homolog was deleted in *Drosophila*, light-induced neurodegeneration of photoreceptors was observed (McQuibban et al., 2006). Finally, in a stroke model, succinate can also induce mitochondrial fission, leading to mitochondrial dysfunction and contributing to neurodegeneration. Thus, mitochondrial dynamics must be tightly regulated to sustain neuronal health, but a role in photoreceptor survival has yet to be addressed (Wu et al., 2017).

#### Dissecting the Impact of Diet on Retinal Health

While links between mitochondrial morphology and metabolic fuel selection is now well established, how individual nutrients influence photoreceptor health through changes in mitochondrial morphology is an important future area of consideration. For instance, fuel selection influences T lymphocyte fate selection—transitioning from glucose to fatty acid usage correlates with increased mitochondrial fusion and the generation of memory T cells (Buck et al., 2016; Raud et al., 2018). In addition, a high fat diet triggers mitochondrial fission in microglia in the hypothalamus, leading to microglial activation and an inflammatory response that precedes weight gain (Kim et al., 2019). Notably, recent studies have demonstrated that the equivalent of a Western diet (i.e., high fat, fructose and glucose) induced metabolic syndrome, with characteristic liver inflammation and fibrosis, as well as hallmark features of AMD (Roddy et al., 2020). How dietary changes impact retinal health at the mechanistic level should be further explored as this study did not directly investigate mitochondrial or other metabolic effects of the dietary changes in the retina (Roddy et al., 2020).

## Metabolism and Photoreceptor Development

### A Primer on Photoreceptor Development

**FIGURE 8:**
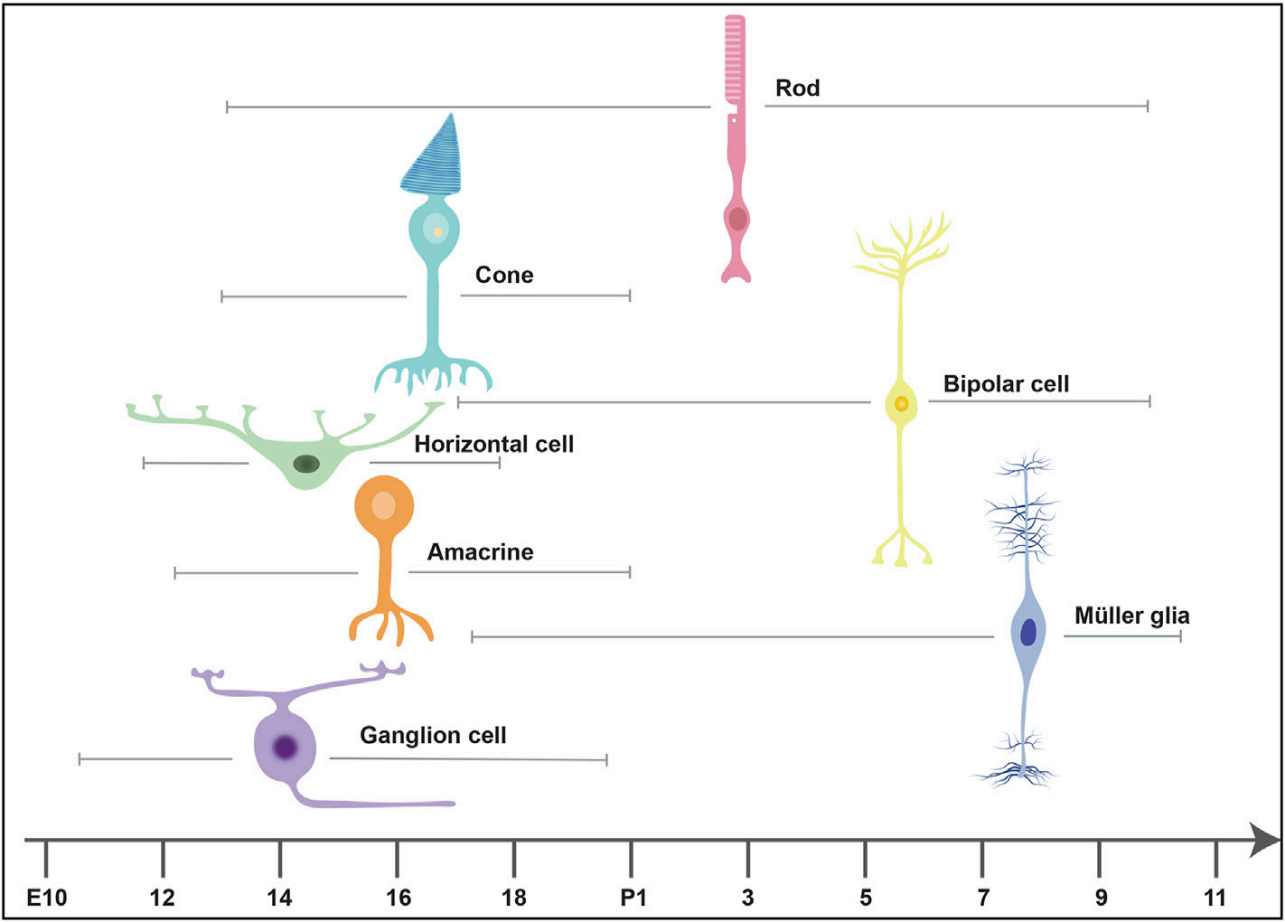
Timing of retinal cell differentiation in the murine retina. The seven retinal cell types are generated in overlapping waves. The embryonic wave involves the sequential but overlapping differentiation or ganglion cells, amacrine cells, horizontal cells and cones, which are born between embryonic day (E) 10 and postnatal day (P) 2. The postnatal wave includes the differentiation of rods, bipolar cells and Müller glia.

**FIGURE 9:**
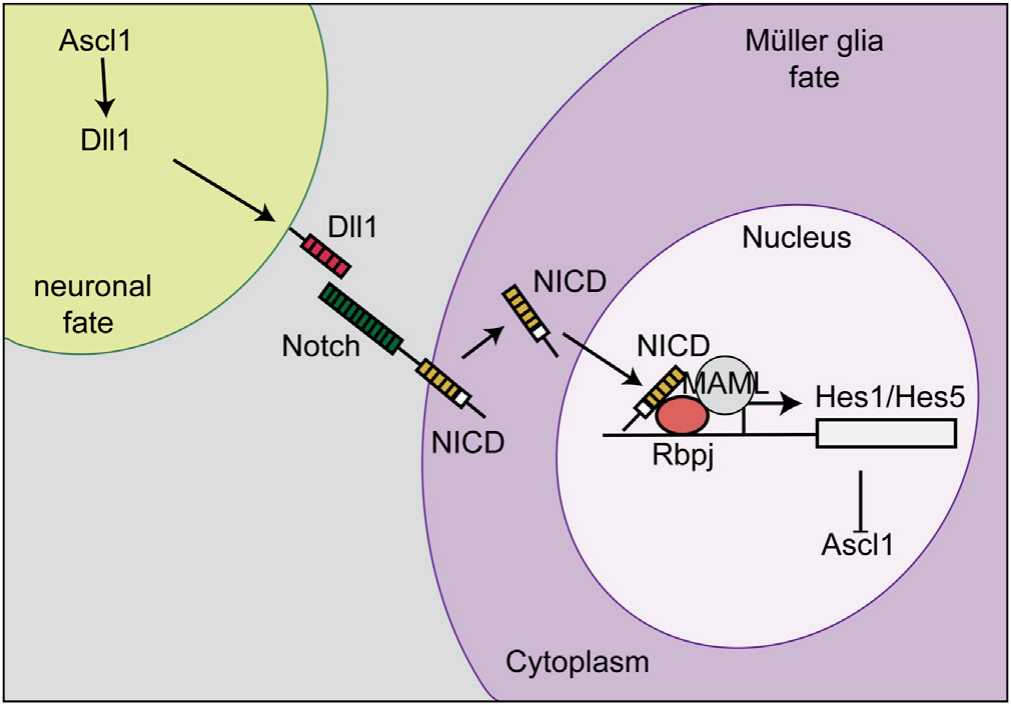
Notch signaling and lateral inhibition. Notch signaling sustains RPC proliferation and prevents differentiation *via* its downstream effectors, Hes1 and Hes5. The cell that becomes a Müller glia expresses high levels of the Notch receptor, which is stimulated by a Delta ligand (Dll1) on the neighboring cell. The Notch intracellular domain (NICD) is then cleaved, translocates to the nucleus, and binds Rbpj to initiate the transcription of *Hes1* and *Hes5*. *Hes1* and *Hes5* repress the transcription of *Ascl1*. This process initiates Müller glia specification and represses differentiation, including a photoreceptor fate.

The Competence Model of retinal development states that retinal progenitor cells (RPCs) are multipotent, and undergo temporal identity transitions to give rise to the seven retinal cell types in a defined, yet overlapping sequence (Javed and Cayouette, 2017). RPCs give rise to most early-born retinal cell types (ganglion, cone, horizontal, amacrine) in the embryonic period, and late-born cell types (rods, bipolar cells, Müller glia) postnatally (Figure 8) in the mouse retina (Javed and Cayouette, 2017). Temporal competence is conferred by the evolutionarily conserved TFs, Ikzf1, and Casz1, homologs of *Drosophila hunchback (hb)* and *castor (cas)*, which direct the correct timing of early-born and late-born cell differentiation, respectively (Elliott et al., 2008; Mattar et al., 2021). The decision by RPCs to proliferate or differentiate is regulated by multiple signaling pathways, including the Notch pathway (Mills and Goldman, 2017). In general, Notch signaling sustains RPC proliferation and prevents differentiation *via* its downstream effectors, Hes1 and Hes5, TFs that inhibit the ability of neural-specific bHLH TFs to induce neurogenesis (Mills and Goldman, 2017) (Figure 9). Sustained Notch activation in murine RPCs thus specifies a Müller glial cell fate (Jadhav et al., 2006a), and specifically inhibits photoreceptor differentiation (Jadhav et al., 2006b; Yaron et al., 2006).

Once RPCs make the decision to exit the cell cycle and differentiate, cell fate choice is at least in part stochastic (Gomes et al., 2011), but is biased by the repertoire of TFs that are expressed. For instance, RPCs that are biased to produce a photoreceptor in a terminal division express the TF Olig2, with early Olig2^+^-RPCs producing cones, and late Olig2^+^-RPCs generating rods (Hafler et al., 2012; Javed and Cayouette, 2017). Olig2 thus confers a generic photoreceptor precursor fate on RPCs (Swaroop et al., 2010), which become cones in the absence of the TF Nrl, and rods when Nrl is present. In turn, Nrl transactivates downstream rod genes, such as *Nr2e3* (Mears et al., 2001; Cheng et al., 2004; Oh et al., 2008). Several other TFs have been identified that are involved in photoreceptor fate specification and differentiation, including Otx2, which is expressed in RPCs with the potential to become photoreceptor precursors (Nishida et al., 2003; Koike et al., 2007). Otx2 initiates expression of Crx, a pan-photoreceptor TF that controls the expression of genes involved in phototransduction (Furukawa et al., 1997; Furukawa et al., 1999). Thus, while the stepwise influence of genetic regulators on cell fate determination in the retina is well understood, the details of how metabolic status directs cell fate specification remain unclear.

### Retinal Metabolism and RPC Proliferation

#### Glycolysis Is the Major Bioenergetic Pathway in RPCs

In general, neural progenitor cells in the embryonic brain favor glycolysis to produce ATP, while differentiated neurons use OXPHOS (Khacho and Slack, 2018). A similar scenario plays out during retinal development, as revealed by the more pronounced drop in ATP production that is observed in differentiated retinal cells versus RPCs when NaN_3_ is used to block complex IV of the electron transport chain (Agathocleous et al., 2012). Notably, glycolysis in RPCs is preferentially fueled by glucose generated from glycogen stores (and not extracellular glucose), as demonstrated by the combined treatment of frog retinas with NaN_3_ and glycogen phosphorylase inhibitor (GPI) resulting in a greater decline in ATP production compared to NaN_3_ and 2-deoxy-glucose (2DG) treatments (Agathocleous et al., 2012). As a consequence of a glycolytic block from the co-administration of 2-DG and GPI, *Xenopus* RPCs reduce their proliferation rates and undergo apoptosis, even though ATP levels remain steady due to compensatory OXPHOS (Agathocleous et al., 2012). Glycolytic metabolism thus has additional roles in supporting RPC mitotic capacity and survival that are unrelated to energy production.

#### mTORC1 Signaling Regulates Glycolysis in RPCs

Glycolytic metabolism is directly regulated by mTORC1 signaling, including in the retina. In RPC-specific *Tsc1* cKOs, glycolytic gene expression is transcriptionally elevated by the TF hypoxia-induced factor 1-alpha (Hif1a) (Lim et al., 2021). In *Tsc1* cKOs, RPC cell cycle length is shortened, and more RPCs actively divide (Choi et al., 2018), an increased proliferative response that is triggered by increased glycolysis as it can be suppressed by 2DG (Lim et al., 2021). Strikingly, the early proliferative expansion of *Tsc1* cKO RPCs is followed by accelerated mitotic aging (i.e., telomere shortening), and senescence-associated cell death, indicating that RPCs have a limited mitotic life-span or division limit (Lim et al., 2021). However, the hyper-proliferation of *Tsc1* cKO RPCs was only observed when *Tsc1* was removed from a small subset of RPCs in the ciliary margin (*Tyrp1-Cre*), and not when *Tsc1* was deleted more broadly in RPCs using *Chx10-Cre* or *Rax-Cre* (Lim et al., 2021). These differences suggest that *Tsc1* cKO RPCs undergo competitive clonal expansion when surrounded by wild-type RPCs, ultimately leading to *Tsc1* cKO RPCs reaching their division limit more quickly. This competitive scenario is not observed when RPCs are all equally capable of dividing. Similarly, *Pten* cKO RPCs generated with a *Pax6-Cre* driver that is only active in peripheral RPCs upregulate mTORC1 signaling and outcompete their wild-type neighbors, leading to the formation of a shrunken central hamartoma comprised of wild-type cells (Tachibana et al., 2018). Additional defects in the timing of retinal cell differentiation are also observed in RPC-specific *Pten* cKO retinas (Cantrup et al., 2012; Jo et al., 2012; Sakagami et al., 2012; Tachibana et al., 2016; Tachibana et al., 2018).

In addition to mTORC1 signaling being sufficient to promote RPC proliferation, there is also a requirement for mTORC1 signaling to sustain RPC division and prevent precocious differentiation (Choi et al., 2018; Jones et al., 2019). Raptor (Rptor) is an adaptor protein that along with mTOR and other subunits makes up the mTORC1 complex. In RPC-specific *Rptor* cKOs generated with *Lhx2-Cre* (Jones et al., 2019) or *Chx10-Cre* (Choi et al., 2018), mTORC1 signaling is reduced and RPC proliferation rates decline, with a change in the timing of ganglion cell differentiation also observed in the *Lhx2-Cre* study. Thus, signaling pathways that regulate metabolism are central determinants of cell fate decisions during retinal development.

### Retinal Metabolism and RPC Differentiation

#### Metabolic Reprogramming During RPC Differentiation

Programmed mitophagy, or the directed autophagy of mitochondria, occurs in several developing tissues to drive metabolic reprogramming from OXPHOS to glycolysis (Ney, 2015). In the retina, there is a striking reduction in mitochondrial mass in the retina during the period when the first ganglion cells are beginning to differentiate (Esteban-Martínez et al., 2017). During embryonic development, when the first wave of RPCs is differentiating, glycolytic metabolites were detected at higher levels in the retina than at postnatal stages, and extracellular acidification rate, a proxy measure of glycolysis, is also elevated (Esteban-Martínez et al., 2017). Strikingly, drugs that inhibit mitophagy prevent the developmental loss of mitochondria, indicating that these organelles are actively targeted for mitophagy in the embryonic retina (Esteban-Martínez et al., 2017). Moreover, by preventing mitophagy, an increase in glycolytic gene expression is no longer observed, indicating that metabolic reprogramming to a glycolytic phenotype requires the programmed destruction of fragmented mitochondria (Esteban-Martínez et al., 2017). This block in mitophagy furthermore reduces ganglion cell differentiation, an effect that is phenocopied by blocking glycolysis with glycolytic inhibitors. Thus, mitochondria must be cleared during early embryonic development to allow ganglion cells to differentiate properly (Esteban-Martínez et al., 2017).

### Future Directions

#### Link Between Metabolism and the Gene Regulatory Networks That Control RPC Differentiation

A future avenue that remains to be explored is how glycolytic pathways intersect with the transcriptional cascades that are known to drive cell differentiation and cell fate specification in the retina. For instance, a critical determinant of an RGC fate is the TF Atoh7, which has a conserved role across species. In chick retina, Atoh7 transactivates Hes5.3 in RPCs that become committed to a ganglion cell fate (Brodier et al., 2021). Strikingly, mitochondrial number is reduced in Hes5.3 expressing RPCs when RGCs begin to differentiate, correlating with reduced PGC1-α expression, a TF that drives mitochondrial biogenesis (Brodier et al., 2021). Accordingly, the authors propose that reduced mitochondrial activity may slow down the cell cycle long enough for Atoh7 to induce a ganglion cell fate (Brodier et al., 2021). This model is in accordance with the cell cycle length hypothesis, which states that progenitor cells poised to differentiate have longer cell cycles so that exposure times to lineage-specifying TFs are increased (Dalton, 2015). To test this model, Brodier et al. (2021) treated chick retinas with FCCP, a mitochondrial uncoupler that binds to protons and equilibrates them across the IMM, thereby dissipating the proton gradient (Brodier et al., 2021). The resulting mitochondrial uncoupling not only increases RPC cell cycle length, but also increases the number of RPCs expressing high levels of Atoh7, a readout of ganglion cell fate commitment (Brodier et al., 2021). Notably, in this chick study, mitophagy is not detected (Esteban-Martínez et al., 2017). Thus, even though fewer mitochondria are required for ganglion cell differentiation in both chick and mouse, the two species use different methods to achieve the same result (i.e., reduced biogenesis in chick versus increased mitophagy in mouse).

Metabolic dynamics may also more directly regulate retinal gene expression by altering chromatin accessibility. Indeed, several major metabolites are essential co-factors for chromatin modifying enzymes. Notable examples include *a*-ketoglutarate, a co-factor for histone demethylases, which play a key role in maintaining embryonic stem cell pluripotency (Carey et al., 2015). In addition, S-adenosyl-methionine is a methyl donor for histone and DNA methyltransferases, and NAD + activates SIRT1 histone deacetylase activity in skeletal muscle stem cell to activate myogenic gene expression (Ryall et al., 2015). Metabolic enzymes also can directly regulate chromatin structure–for instance, acetyl-CoA synthetase translocates to the nucleus and regulates histone acetylation in the hippocampus to consolidate spatial memory (Mews et al., 2017). A comprehensive analysis of chromatin accessibility and modifications during RPC differentiation will allow investigators to elucidate the link between metabolic dynamics, gene expression and chromatin structure during retinal development.

#### Impact of Accumulating Metabolites, Such as Lactate and Succinate, on RPC Differentiation

During glycolysis, accumulated pyruvate is converted to lactate in the cytosol and exported out of the cell. Lactate has long been thought to be a simple waste product, but there is growing support for the idea that lactate participates in cell-to-cell communication (Nasu et al., 2021). For instance, in the nervous system, lactate released by astrocytes is taken up by neurons to fuel the TCA cycle in what has been termed an astrocyte-to-neuron lactate shuttle. New genetic tools, such as the eLACCO1.1 biosensor (Nasu et al., 2021), have been developed that would allow to trace lactate transfer in the developing retina, which may provide novel insights into how lactate production by RPCs may fuel energy demands of differentiating retinal cells, including photoreceptors. Another TCA cycle intermediate is succinate, which accumulates in low O_2_. Interestingly, by binding an orphan G-coupled receptor, succinate can induce retinal angiogenesis in response to hypoxia (Sapieha et al., 2008). Future work to delineate the role of metabolites like lactate and succinate and their transfer between cells in retinal development will be of value to the field.

#### Impact of Lactate-Driven Changes in Intracellular pH in Driving Retinal Cell Differentiation

When lactate is exported out of the cell by lactate/H^+^ symporters (Slc16a1 family genes), it is transported along with one H^+^. The net effect is lower extra-cellular pH (pHe) and higher intra-cellular pH (pHi) (Oginuma et al., 2020). Consequently, when glycolysis increases, so too does pHi. Tissues naturally have pHi and differentiation gradients–e.g., there is an anterior (low pHi, more differentiated cells) to posterior (high pHi, more progenitor cells) gradients in the embryonic chick tailbud (Oginuma et al., 2020). Future experiments could determine whether similar pH gradients exist in the developing retina using ratiometric pH sensors, such as pHluorin, which has a bimodal activation spectrum—higher 488/405-nm ratio indicates lower pHi. Hence, the consequences of altered pH on retinal development could be investigated using these molecular probes. For instance, lowering pHi biases neuromesodermal progenitor cells to a neural fate in the embryonic chick tailbud, acting in part by promoting enzyme-independent β-catenin acetylation to activate Wnt signaling (Oginuma et al., 2020). Notably, Wnt signaling inhibits RPC differentiation and sustains a proliferative RPC phenotype by inhibiting the expression of the proneural bHLH TFs (Kubo et al., 2005). Whether Wnt or other signaling pathways are similarly influenced by elevated glycolysis in the retina, which sustains RPC proliferation, is an exciting open question that once answered will uncover a new level of signaling pathways regulation. A future focus may be to investigate glycolysis modulation as a novel way to direct cell fate in retinal regenerative strategies as an alternative to targeting the signaling pathways directly.

#### Impact of mTOR/Glycolysis Axis on mRNA Translation and Protein Synthesis

The gene regulatory networks (GRNs) that underlie cell fate decisions in the developing retina are beginning to be deciphered with the use of single cell transcriptomic and open chromatin analyses (Lyu et al., 2021). However, many transcripts expressed in the developing retina are not translated or translated with a delay, especially before eye opening (Chen et al., 2021). Interestingly, mRNA translation is regionally localized, with late endosomes serving as translation hubs in growing retinal ganglion cell axons (Cioni et al., 2019). Strikingly, as these translation hubs move towards the growth cone, they often pause on mitochondria they encounter, which serve as focal hotspots of translation (Cioni et al., 2019). Given that mTOR signaling regulates protein translation by inducing mRNA interactions with eukaryotic translation initiation factor 4F (eIF4F), rRNA synthesis, and the subsequent recruitment of ribosome subunits (Nandagopal and Roux, 2015), it seems plausible that regulation of mTOR might control when and where mRNA is translated in the developing retina. Establishing where mTOR is localized during axonal development, and how this localization guides mTOR function and possibly mRNA target selection will be important questions to address.

In *Drosophila*, mTOR signaling was shown to facilitate photoreceptor differentiation by downregulating *Unkempt* (*Unk*), a zinc finger/RING domain protein that acts as negative regulator of photoreceptor differentiation (Avet-Rochex et al., 2014). One of the critical ways in which mTORC1 mediates downstream effects is by activating ribosomal S6 kinases (P70S6K), which phosphorylates 4E-BP1, thereby removing inhibitory controls on eukaryotic initiation factor 4E (eIF-4E), a central regulator of cap-dependent translation (Averous and Proud, 2006). Accordingly, elevated Hif1a levels in *Tsc1* cKO RPCs have been attributed in part to increased translation (Lim et al., 2021). Conversely, the increase in cell cycle proteins that are observed in *Tsc1* cKO RPCs persists even after rapamycin treatment, an mTORC1 inhibitor. Instead, mTORC1 activation promotes more rapid cyclin degradation, which likely accounts for increased oscillations of these proteins to facilitate the enhanced rate of cell cycle progression in *Tsc1* cKO RPCs (Choi et al., 2018). These studies highlight the need to further dissect apart how metabolic signaling pathways regulate retinal development. Understanding how mTORC1 regulates neuronal differentiation, either *via* either translation or protein degradation will be an exciting future area of investigation.

#### Impact of Mitochondrial Dynamics and ROS Levels on Retinal Differentiation

In dormant stem cells, mitochondria fuse and form elongated networks, whereas they undergo fission (fragmentation) following stem cell activation (van Velthoven and Rando, 2019). Such mitochondrial shape changes, or dynamics, drive stem cell transitions–e.g., increased fission induces muscle stem cell proliferation and differentiation (Khacho et al., 2014; Khacho et al., 2016; Khacho et al., 2019). In brain neural stem cells, mitochondrial fission directs cells towards a committed fate, while fusion directs stem cell self-renewal (Khacho et al., 2016). Investigating the role of mitochondrial fission and fusion in balancing RPC proliferation and differentiation is an important future area of investigation. Indeed, as mitochondria are important hubs for integration of numerous signaling pathways, they have roles in cell physiology outside of production of ATP, central among which is the production of metabolic intermediates to generate biomass. In this regard it is interesting that mitochondrial mass is reduced in RPCs during early embryonic development, when ganglion, horizontal, cone and amacrine cells are forming, but not during the postnatal period, when rod photoreceptors, bipolar cells and Müller glia are being generated (Esteban-Martínez et al., 2017). This finding raises the question of whether mitochondria play an important role in guiding later stage differentiation decisions, and also whether late RPCs like early RPCs use glycolysis for energy production. Interestingly, mitochondrial ROS has been proposed to act as a rheostat, with small dynamic changes in ROS levels regulating hematopoietic stem cell proliferation and differentiation decisions, while larger increases in ROS can trigger pathogenic reactions such as apoptosis (Maryanovich and Gross, 2013).The biology of ROS signaling–including how it is generated, detected, and acted upon at the molecular level—is an untapped area of investigation in the field of retinal development.

#### Impact of Lysosomes, the Forgotten Metabolic Organelle, on RPC Proliferation and Differentiation

As introduced above, lysosomes degrade unwanted proteins and organelles in a regulated manner to maintain cellular homeostasis, but also play an underappreciated role as metabolic sensors (Todkar et al., 2017). In this capacity, lysosomes detect starvation cues that promote stem cell quiescence, as observed in the hematopoietic system (García-Prat et al., 2021a). The switch between hematopoietic stem cell quiescence and activation is governed by a balance between lysosomal and mitochondrial biogenesis, mutually exclusive events: when one is up, the other is down (Todkar et al., 2017). These two events are regulated by two opposing transcription factors, Myc and Tfeb (García-Prat et al., 2021a), which compete for the same binding sites in the genome with opposing effects (Annunziata et al., 2019). mTORC1 promotes mitochondrial biogenesis by activating Myc (de la Cruz López et al., 2019), and suppresses lysosomal biogenesis by phosphorylating and sequestering Tfeb in the cytosol (Martina et al., 2012; Settembre et al., 2012). The importance of the lysosome in stem cell maintenance was further supported by a CRISPR-screen that identified several lysosomal genes that regulate in embryonic stem cell differentiation (Villegas et al., 2019). Lysosomal activation also correlates with the differentiation of neural stem cells into specific cell fates (Delaney et al., 2020). Thus, further investigation of how lysosomal biogenesis and function regulates the choice by multipotent retinal progenitor cells to differentiate into specific cell fates is worth investigating.

## Endogenous Retinal Regeneration: Targeting the Latent Repair Capacity of Müller Glia

### Müller Glial Structure and Function

Müller glial cells are well positioned to have myriad roles in maintaining tissue homeostasis as they span almost the entire retinal thickness and ensheathe all retinal cell types. Müller glia have bidirectional processes that terminate with endfeet that form two barriers–an inner (ILM) and outer (OLM) limiting membrane (Varshney et al., 2015). Based on these features, it has been proposed that Müller glia act like springs to hold the retina together. Accordingly, the active tensile properties of Müller glia are required to maintain an intact retina, as their deletion from the zebrafish retina results in retinoschisis, a blinding disease in which the retina splits into two layers (MacDonald et al., 2015).

The ILM is a true basement membrane as it is associated with an extracellular matrix (ECM) that is made up of laminins, type IV collagens and other ECM proteins. While the main role of the ILM is to separate the neural retina from the vitreous, ILM connections to RPCs also guide their differentiation, while ILM connections to ganglion cells facilitate the establishment of axonal and synaptic contacts (Varshney et al., 2015). In contrast, the OLM is not a true basement membrane, even though it does have barrier properties. The OLM is formed by interconnected Müller glia endfeet that form adherens junctions with photoreceptors to create a semi-permeable barrier that prevents the diffusion of larger proteins/molecules (Omri et al., 2010). Disruption of the OLM is associated with pathological features of certain diseases, such as the macular edema observed in patients with diabetic retinopathy, highlighting essential OLM barrier functions (Omri et al., 2010). Notably, even when a gliotic response is observed in mouse models of degeneration, the OLM is not necessarily disrupted. For instance, while OLM disruption was observed early in the degeneration process in *Crb1* ^*rd8/rd8*^, *Prph2* ^*+/307*^, and *Pde6b* ^rd1/rd1^ mutant mice, the *Rho* ^*−/−*^ mouse model of RP showed disruption of OLM only in late stages of the disease (Hippert et al., 2015).

Additional Müller glia functions include the regulation of ion, neurotransmitter, and metabolite exchange between retinal neurons and external compartments such as blood vessels, RPE and vitreous humor (Reichenbach and Bringmann, 2013). For instance, Müller glia buffer extracellular potassium *via* Kir channels, transport water through AQP4 channels, and clear CO_2_ through carbonic anhydrase activity (Reichenbach and Bringmann, 2013). Müller glia also play a key role in the production of neurotransmitter precursors, catalyzing the conversion of glutamate to glutamine by glutamine synthetase, which is then used by photoreceptors to remake glutamate as neurotransmitter to relay signals to bipolar cells (Pow and Crook, 1996). Müller cells also recycle glutamate and GABA neurotransmitters, and are well positioned to carry out these functions as they extend processes in the plexiform layers that ensheathe neuronal synapses (Salman et al., 2021).

### Müller Glial Regenerative Capacity

**FIGURE 10:**
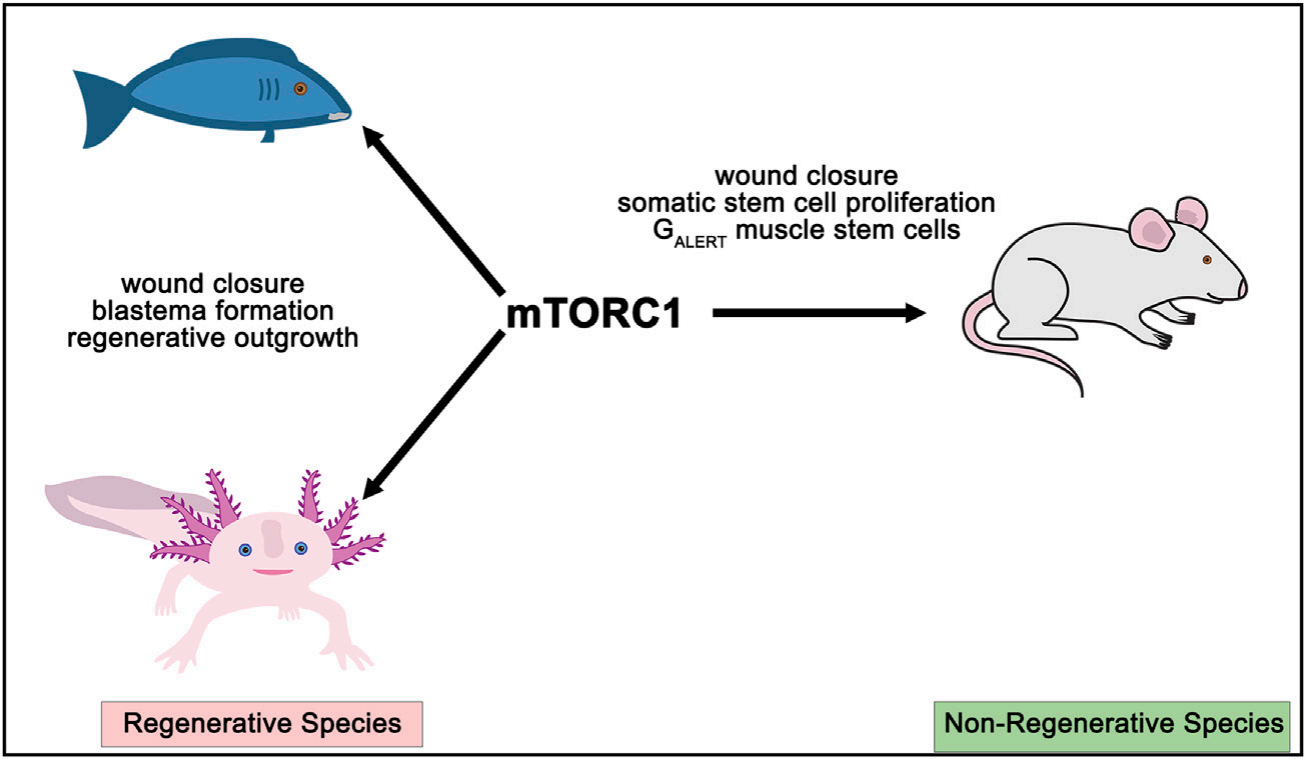
Central position of mTORC1 as a critical regulator of tissue repair. mTORC1 drives wound closure, blastema formation and regenerative outgrowth, including in the retina, in teleost fish and the axolotl, both considered regenerative species. In non-regenerative species, such as mammal, mTORC1 signaling is also important for wound closure, somatic stem cell proliferation, and for the initial activation of dormant stem cells in skeletal muscle to a G_ALERT_ phase.

**FIGURE 11:**
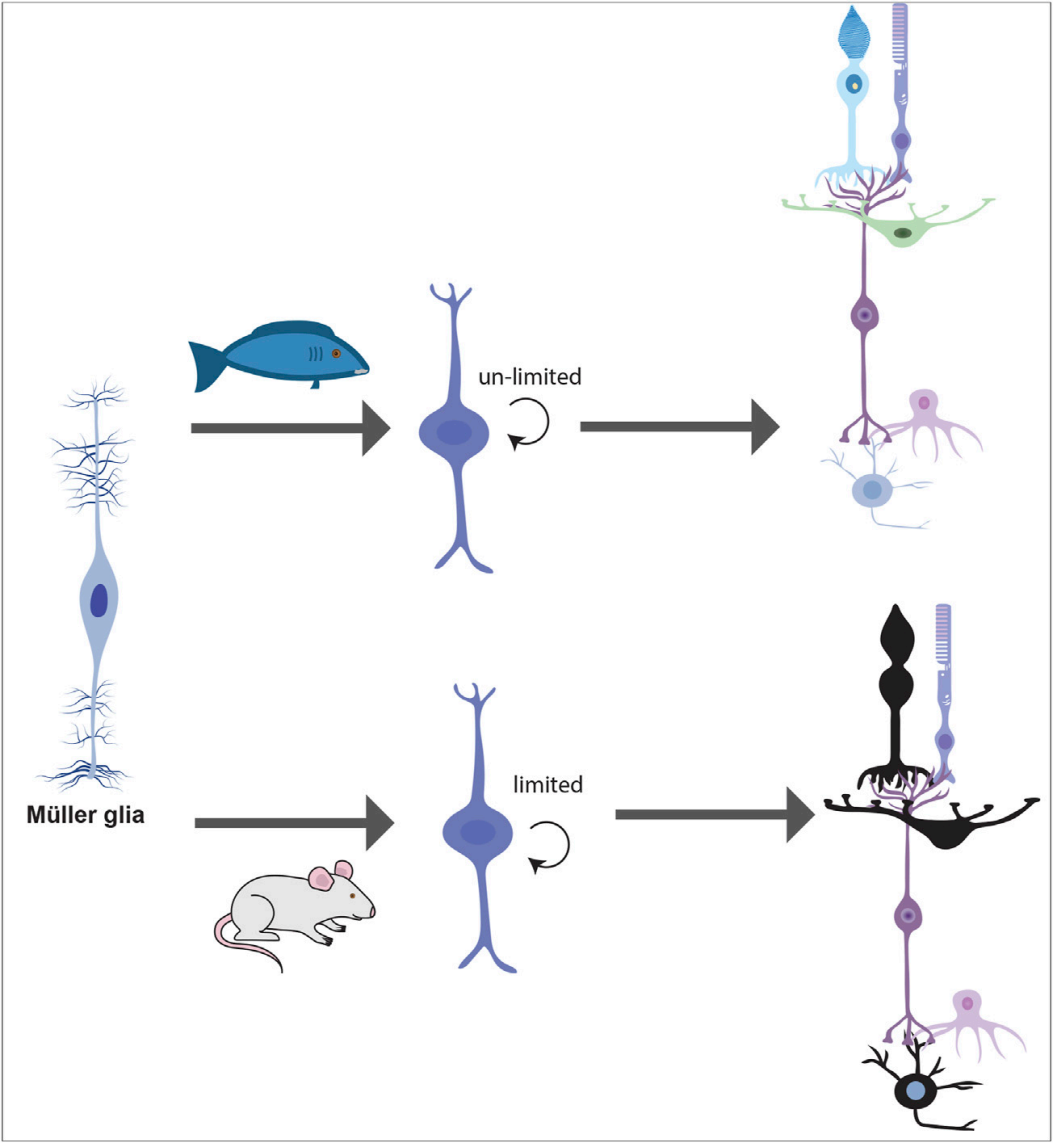
Müller glia regeneration in fish versus mice. In regenerative species such as teleost fish, Müller glia de-differentiate into Müller glial progenitor cells (MGPCs) that have an unlimited capacity to divide. These MGPCs can give rise to all seven mature cell types in the retina. In non-regenerative species such as rodents, Müller glia have a very limited capacity to divide, and when they are stimulated to give rise to new cells (by injury, growth factors and Ascl1), they give rise to only a limited number of mature retinal cell types. This “mammalian regenerative” capacity is very limited.

All animals have some capacity to regenerate lost body parts, but this ability varies greatly between species and within individual tissues and organs. Regenerative species have a robust wound response governed by mTORC1 that allows them to regenerate entire appendages, such as fins in zebrafish and limbs in axolotl (Johnson et al., 2018) (Figure 10). However, when neurons in the mammalian retina are lost due to injury or disease, like those in the brain, they are not replaced (Blackshaw and Sanes, 2021). Intriguingly, teleost fish possess the natural ability to readily regenerate an injured retina (Wan and Goldman, 2016). The teleost regenerative response relies on adult Müller glia, which have the remarkable capacity to respond to injury by de-differentiating, entering the cell cycle to proliferate as Müller glial progenitor cells (MGPCs) and then re-differentiating into any of the lost cell types, including photoreceptors (Figure 11) (Goldman, 2014). Of note, the ability of zebrafish Müller glia to respond to injury persists throughout life, even though these animals undergo neuronal degeneration with aging that is not repaired (Martins et al., 2022).

In general, mammalian Müller glia lack the same regenerative response to injury, instead undergoing reactive gliosis, which at early stages limits the spread of injury and confers needed neuro-protection (Bringmann et al., 2009). Notably, zebrafish Müller cells also become gliotic as the initial response to injury, but then transit to the proliferative phase of repair, as described (Thomas et al., 2016). In mammals, prolonged gliosis has detrimental effects, leading to edema, formation of glial scars and neurodegeneration (Bringmann and Wiedemann, 2012). Strikingly, a recent comparison of murine and fish Müller glia transcriptomic and epigenomic profiles found that both species mount the same initial response to injury, but in mice, the activated state is rapidly aborted, and does not progress to the proliferative and neurogenic phases of repair (Hoang et al., 2020).

Several signals have been identified that can stimulate Müller glia to proliferate, including injury itself and growth factor signaling, with ectopic Wnt (Liu et al., 2013), Notch (Del Debbio et al., 2010), or Hedgehog (Wan et al., 2007) signaling, or inhibition of Hippo signaling (Hamon et al., 2019), driving at least some level of proliferation by mammalian Müller glia. However, even when N-methyl-d-aspartic acid (NMDA)-injured retinas are supplemented with exogenous epidermal growth factor (EGF), only 1% of Sox2-labelled Müller glia entered S-phase and incorporated BrdU, demonstrating the rate-limiting response even when injury and growth factors are present (Karl et al., 2008). Another key to the puzzle is the proneural gene *Ascl1* (Ueki et al., 2015)*,* which encodes a rate-limiting TF for Müller glia regeneration (Fausett et al., 2008). *Ascl1* is not naturally expressed in adult mammalian Müller glia but upregulated in fish and avian Müller glia following injury (Karl and Reh, 2010). When *Ascl1* is introduced into mammalian Müller glia in the early postnatal period following retinal insult, Ascl1 triggers some regenerative events, including limited glial trans-differentiation into retinal neurons (Karl et al., 2008; Karl and Reh, 2010). However, for *Ascl1* to have similar functions after injury beyond the post-natal period, treatment with histone deacetylase (HDAC) inhibitors is required for efficient reprogramming of Müller glia (Jorstad et al., 2017; Hoang et al., 2020). Finally, while there are contradictory reports on the ability of various genetic manipulations to trigger mammalian Müller glia to give rise to new retinal cells, either by first de-differentiating and proliferating as progenitor cells, or directly transdifferentiating (outside of the scope of this review), the limited capacity of these glial cells to naturally repair the retina in mammals is universally agreed upon (Blackshaw and Sanes, 2021).

### Müller Glia Use Glycolysis as the Main Energy-Producing Metabolic Pathway

Current dogma suggests that adult Müller glia preferentially use aerobic glycolysis as the main energy producing pathway (Winkler et al., 2000). Indeed, *in vivo* imaging in Müller glia using fluorodeoxy-d-glucose (FDG) PET imaging have shown glucose metabolism through the classical glycolytic pathway (Poitry-Yamate et al., 2013). Moreover, treating human Müller glia in culture with iodoacetate, a glycolytic inhibitor, lowered ATP production whereas mitochondrial inhibitors had little effect on ATP levels (Winkler et al., 2000). Glycolysis is also important for Müller glial survival, as these cells undergo apoptosis in response to glycolytic inhibitors, but can survive in the absence of glucose (due to the presence of glycogen stores) or in reduced O_2_ (Winkler et al., 2000). Interestingly, despite their reliance on glycolysis, Müller glia do not produce all of the needed glycolytic enzymes, and instead form a symbiotic relationship with neighboring photoreceptors (Lindsay et al., 2014). Indeed, murine Müller glia lack pyruvate kinase, which catalyzes the last step in the glycolytic enzyme chain to generate pyruvate (Lindsay et al., 2014). In the absence of pyruvate, Müller glia fuel their mitochondria by taking up lactate from photoreceptors, and then oxidizing lactate to pyruvate (Lindsay et al., 2014). Müller glia also do not express an aspartate/glutamate carrier 1 (AGC1), but a second compensatory pathway allows these glial cells to take up aspartate from photoreceptors, which is then used to fuel the generation of glutamine (Lindsay et al., 2014). These symbiotic relationships thus allow Müller glia to fuel their mitochondria with photoreceptor-derived metabolites.

### Role of Müller Glia Mitochondria

While glycolysis in Müller glia is of central importance across the retina, recent studies have revealed the contributions of mitochondrial OXPHOS. Müller glia mitochondria are more plentiful and evenly distributed in vascularized retinas, such as in human and mice, where oxygen levels are relatively constant throughout the apico-basal layers. In contrast, in retinas of avascularized mammals like rabbit and guinea pig, Müller glia mitochondria are scarce and restricted to regions of higher O_2_ concentrations (Germer et al., 1998). Thus, mitochondria likely play a pivotal role in maintaining energy homeostasis in vascularized Müller glia. In this regard, healthy Müller glia may require proper mitochondrial function for fine-tuning glutamate uptake and conferring neuroprotection to retinal neurons (Toft-Kehler et al., 2017).

Glutamate is the primary excitatory neurotransmitter in the retina and is released by photoreceptors, bipolar and ganglion cells. However, excess glutamate can cause excitotoxic damage to neurons, leading to irreversible damage and cell death, a feature of ganglion cell death in glaucoma (Lotery, 2005). Müller glia bear the pivotal responsibility of removing glutamate from the retinal synapse to confer neuroprotection and to modulate phototransduction (Holcombe et al., 2008). The major glutamate transporter in Müller glia is GLAST (glutamate-aspartate transporter), an ATP-dependent process that removes more than 50% of retinal glutamate from the synapse (Sarthy et al., 2005). However, for GLAST and other related active glutamate transporters to efficiently work, substantial amounts of ATP are required. Thus, in cases where Müller glia mitochondria have been impaired, glutamate accumulates in the synapse and triggers neuronal cell death (Toft-Kehler et al., 2017).

The second role of Müller glia mitochondria is the catabolism of glutamate by the TCA cycle (tricarboxylic acid cycle). Here, Müller glia mitochondria express glutamate dehydrogenase (GDH) which catalyzes the conversion of glutamate to *a*-KG, an intermediate of the TCA cycle (Ola et al., 2011). The oxidative deamination of glutamate thus supplies carbons to the TCA cycle, a process known as anaplerosis (Ola et al., 2011; Toft-Kehler et al., 2017). The use of glutamate to fuel the TCA cycle in mammalian Müller glia suggests that glutamate may be an alternative energy source. Emerging data have shown that glutamate uptake by Müller glia increases in times of hypoglycemia, meaning that Müller glia adapt to the change in available energy source to maintain retinal homeostasis (Toft-Kehler et al., 2017). Such contributions of Müller glia mitochondria emphasize the important role of OXPHOS in conditions of altered metabolic demands (Toft-Kehler et al., 2017). Thus, it has been postulated that increasing the function of Müller glia mitochondria may confer neuroprotection during retinal pathologies such as glaucoma and diabetic retinopathy.

### Future Directions

#### Harnessing the Regenerative Capabilities of Muller Glia by Targeting mTOR and Glycolytic Metabolism

To understand the inability of mammalian Müller glia to regenerate, clues may be found in comparisons to stem cell regulation in other cell lineages. Stem cells support tissue homeostasis in regenerative species such as fish and in the few mammalian tissues that can regenerate (e.g., skeletal muscle, hematopoietic system). Studies across organisms and species have revealed that somatic stem cells that support regeneration exist in a continuum of states, transiting from deep dormancy to primed quiescence, or G_ALERT,_ before they are activated to proliferate and differentiate (Rodgers et al., 2014; Llorens-Bobadilla et al., 2015; Urbán et al., 2016; Laurenti and Göttgens, 2018; Urbán et al., 2019). G_ALERT_ stem cells are poised to respond to injury, and increase their metabolism (mitochondrial biogenesis and fission, glycolysis) to support a proliferative response (Khacho et al., 2019; Rodgers et al., 2014; Liang et al., 2020; Dell'Orso et al., 2019).

In regenerative species like zebrafish, metabolism lies at the centre of regenerative pathways, inhibiting glucose metabolism with 2DG resulted in the loss of fin regeneration capability (Sinclair et al., 2021). In mammals, mTORC1 expression and activity is highly up-regulated in G_ALERT_ stem cells in the skeletal muscle of the contralateral limb, compared to quiescent stem cells in an unperturbed animal. Using the muscle stem cell-specific *Pax7* ^*CREER*^ driver to ablate *TSC1* (tuberous sclerosis protein 1), a negative regulator of mTORC1, the authors were able to stimulate stem cells to enter G_ALERT_ stem cells from a totally quiescent stem cell populations without any contralateral injury (Rodgers et al., 2014). In contrast, the ablation of *Raptor*, a key component of mTORC1, suppressed mTORC1 signaling and resulted in a complete loss of G_ALERT_ stem cells in the contralateral stem cell niche, even with injury (Rodgers et al., 2014). Along with other experiments, mTORC1 was firmly demonstrated to be necessary and sufficient for G_0_-G_ALERT_ transition, and in the absence of regenerative cues mTORC1 signaling maintains G_ALERT_ stem cells in a primed state ready to respond (Rodgers et al., 2014). mTORC1 drives regenerative processes through the animal kingdom and across tissues (Lund-Ricard et al., 2020), including in chick Müller glia (Zelinka et al., 2016), *via* unknown mechanisms. Given its interplay with metabolic dynamics, understanding how mTORC1 signaling and downstream glycolytic pathways regulates Müller glia activation is critical to the design of future regenerative strategies.

#### Investigating the Role of Metabolic Gene Regulatory Networks in Müller Glia Regeneration

**FIGURE 12:**
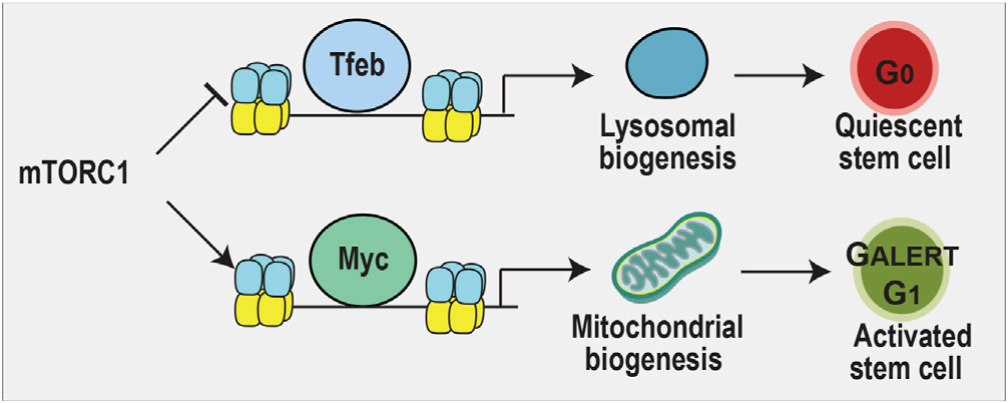
Myc and Tfeb TFs compete for target sites in the genome. Myc induces mitochondrial biogenesis and stem cell activation. Tfeb promotes lysosomal biogenesis and stem cell dormancy. mTORC1 inhibits Tfeb function and promotes Myc activity during tissue regeneration, as shown in the hematopoietic system.

The failure of mammalian Müller glia to regenerate can be attributed to the lack of inductive/permissive signals or/and the presence of inhibitory cues. Most groups focus their effort on studying potential inductive signals that can drive Müller glia to proliferate, which includes the triggering event of the injury itself and the growth factor signaling response. However, what molecular machinery constitutes the brake that transitions mammalian Müller glia into quiescence and thus prevents retinal repair, remains an open question (Hoang et al., 2020). A ground-breaking study identified gene regulatory networks (GRNs) that are associated with a return-to-quiescence program by mammalian Müller glia post-injury (Hoang et al., 2020). However, the importance of GRNs that control metabolic reprogramming have yet to be investigated. Interestingly, in hematopoietic stem cells, lysosomal and mitochondrial biogenesis are mutually exclusive events (i.e., when one is up, the other is down) (Todkar et al., 2017). As detailed above, this switch is governed by a Myc-centered mitochondrial GRN and Tfeb-centered lysosomal GRN (Figure 12). Understanding the contribution of Myc and Tfeb to Müller glia regeneration could provide important new clues to the role of metabolic events in regulating glial cell regenerative capacity.

#### Metabolic Influence on Glia to Neuron Reprogramming

The quest is on to find the TFs and other inducing signals that may trigger Müller glia to act like stem cells and repair the damaged retina, but the importance of metabolic pathways may be under appreciated (Blackshaw and Sanes, 2021). If mitochondrial activation facilitated Müller glia proliferation, conversion of Müller glia to neurons may also be under the control of metabolic programming. Indeed, there is growing evidence that neuronal reprogramming in the brain is influenced by metabolic pathways. For example, *in vivo* reprogramming of glia to neurons is improved when Bcl12 is expressed, but not for the expected anti-apoptotic effects (Gascón et al., 2016). Instead, Bcl2 reduces neuronal reprogramming-associated lipid peroxidation, the blockage of which prevents induced neurons from undergoing ferroptosis. Additionally, a proteomic study revealed striking differences in mitochondrial proteins in neurons versus astrocytes, and the specific expression of neuronal genes improved neuronal reprogramming efficacy (Russo et al., 2021). Given the central role of metabolism, the potential impact of diet and dietary supplements on Müller glia regenerative response has, to the best of our knowledge, not been reported. Interestingly, in zebrafish, in which Müller glia are normally regenerative, Müller glia display a reduced capacity to proliferate and generate new neurons in a genetic model of chronic diabetes (Schmitner et al., 2021). Moreover, altering metabolism in this diabetic model inactivates Notch signaling, an essential component of the regenerative response in the zebrafish retina. It is important to note, however, that metabolic changes associated with chronic disease are not necessarily directly comparable to changes observed in acute injury models (Schmitner et al., 2021). In the rat, a recent study found that high glucose exposure induces oxidative stress in retinal Müller glia *in vitro*, and identified Nrf2 as a critical transcription factor in this response (Albert-Garay et al., 2022). More studies are required to understand how metabolic transitions influence Müller glial cell activation and the repair response.

#### The Effect of Metabolism on Axonal Growth and the Formation of Functional Neuronal Network

Once new neurons have been generated, an important remaining question remains is how required functional connections be established? Indeed, CNS neurons do not regenerate axons after injury. Much of the injury response is dependent on glial cells, which guide axonal growth. Interestingly, in the *Drosophila* CNS, artificially increasing glycolysis in glial cells led to the accumulation of excess metabolites (l-lactate, L-2HG), which acted on GABA_B_ receptors to promote neuronal outgrowth (Li et al., 2020). Moreover, l-lactate could stimulate regrowth of corticospinal axons in a mouse model of spinal cord injury, leading to improved functional outcomes (Li et al., 2020). Thus, metabolism is a central regulator of axonal growth.

In the murine retina, Müller glia can be induced to differentiate into bipolar cell-like neurons by expressing neurogenic bHLH TFs, together with a stimulating injury and HDAC inhibitor (Todd et al., 2021). In another study, Müller glia were successfully converted into functional photoreceptor-like cells in a degenerative model by activating Wnt signaling and overexpressing 3 TFs that promote neurogenesis and photoreceptor fate specification (Yao K. et al., 2018). However, although these photoreceptors were efficient in capturing light, the response recorded from RCGs cells was weak and resembled an unstimulated RCG response in a healthy retina. The authors hypothesized that this weak response could be due to the low number of reprogrammed cells, but other possibilities include incomplete differentiation, leading to aberrant connectivity and synapse formation. Not investigated in these studies is the potential significance of metabolites in guiding axon outgrowth, as highlighted above. Thus, a full understanding of how metabolic pathways are regulated during regenerative events is essential for the future design of cell therapeutics.

## Discussion

Emerging data has revealed that metabolic machinery influences cell function in diverse ways that go beyond simple energy production. Here we have presented recent discoveries that reveal how metabolic pathways influences retinal cell development, photoreceptor cell health and survival, and the prospect of glial-based endogenous repair. We have also highlighted how metabolism may be similarly interrogated or manipulated in the retina to improve cell health, potential diagnostics, and promote tissue repair. While the importance of energy metabolism may be relatively new to investigators who study retinal cell biology, it is important to note that metabolic considerations are rapidly becoming an important area of investigation for investigators studying the regeneration of multiple tissues, including the lung, heart, and liver. Given that hypometabolism during aging can influence brain function and tissue health, and that Western high-fat, high-sugar diets can accelerate degenerative processes, the need to consider the profound effects of metabolism in the design of novel therapeutic strategies for vision disorders is clear.
